# High-Q asymmetrically cladded silicon nitride 1D photonic crystals cavities and hybrid external cavity lasers for sensing in air and liquids

**DOI:** 10.1515/nanoph-2022-0245

**Published:** 2022-08-10

**Authors:** Simone Iadanza, Jesus Hernan Mendoza-Castro, Taynara Oliveira, Sharon M. Butler, Alessio Tedesco, Giuseppe Giannino, Bernhard Lendl, Marco Grande, Liam O’Faolain

**Affiliations:** Tyndall National Institute, Lee Maltings, Dyke Parade, Cork, Ireland; Munster Technological University, Rossa Avenue, Bishopstown, Cork, Ireland; DEI, Politecnico di Bari, Via Amendola 126/b, Bari, Italy; TUW, Institute of Chemical Technologies and Analytics, Getreidemarkt 9/164, 1060 Vienna, Austria

**Keywords:** integrated sensors, laser, nanophotonics, photonic crystals, refractive index sensing, silicon photonics

## Abstract

In this paper we show a novel design of high Q-factor silicon nitride (SiN) 1D photonic crystal (PhC) cavities side-coupled to curved waveguides, operating with both silica and air cladding. The engineering of the etched 1D PhC cavity sidewalls angle allows for high Q-factors over a wide range of upper cladding compositions, and the achievement of the highest calculated Q-factor for non-suspended asymmetric SiN PhC structures. We show the employment of these type of SiN PhC cavities in hybrid external cavity laser (HECL) configuration, with mode-hop free single mode laser operation over a broad range of injected currents (from 25 mA to 65 mA), milliwatts of power output (up to 9 mW) and side-mode suppression ratios in the range of 40 dB. We demonstrate the operation of these devices as compact and energy efficient optical sensors that respond to refractive index changes in the surrounding medium the measurement of sodium chloride (from 0% to 25%) and sucrose (from 0% to 25%) in aqueous solution. In HECL configuration, the RI sensor exhibits a 2 orders of magnitude improvement in detection limit compared to the passive microcavity. We also discuss the possibility for applying these devices as novel transducers for refractive index changes that are induced by analyte specific absorption of infrared radiation by the target analytes present in gas or liquid phase.

## 1 Introduction

In the last few years, the ever-growing investigation of fast and sensitive optical biosensing has led to a wide range of applications ranging from virus and bacteria detection to environmental monitoring of pollutants [1]. Current technologies include: nanoscale sensors, such as nanowires and plasmonic particles, presenting a relatively small capture area, hindering analyte detection [2, 3] and mechanical cantilevers, commonly used for sensing in air or vacuum but not employable in liquids due to oscillation dampening [4]. As refractive index of a liquid is a volume independent quantity, refractive index sensors offer advantages as they are not affected by sample volume, unlike intensity/fluorescence-based sensors, and the signal is not proportional to the sample volume, rendering these sensors highly competitive in sensitivity, especially in lab-on-chip systems [5]. This feature has led to a large number of photonic devices being developed to operate as refractive index sensors. Among the most used optical sensors are nanostructured porous silicon [6–9] and whispering-gallery mode (WGM) optical resonators [], such as micro-disks (MDs) [18–22] and micro-ring resonators (MRRs) [23–26], which offer high speed and sensitivity for label-free sensing, but greatly suffer from challenging on-chip integrability and multiplexing possibility due to their morphology or sizes [27]. An emerging solution for label-free biosensing is presented by optical devices based on photonic crystal (PhC) cavities [28–31], as they are characterized by: (I) very low losses and small footprint [32, 33], but larger capture area compared to nanoscale sensors [34], and (II) flexibility and great on-chip integrability [35, 36], as opposite to WGM optical sensors.

Among the photonic crystal family, one-dimensional (1D) PhC cavities, such as the nanobeam design, are attracting interest because of their high Q-factors, design resiliency, and relatively easy fabrication [37, 38]. Furthermore, in order to exploit the potential of the CMOS industry for the low-cost manufacture of photonic integrated sensors in high volumes, a great effort is being directed to develop sensors on CMOS compatible materials, such as silicon and silicon nitride (SiN). In particular, SiN 1D PhC cavities (nanobeams) offer advantages over their silicon counterparts, such as a broader range of operating wavelengths, spanning from the visible to the near-infrared (NIR) spectrum, and the absence of two-photon absorption (TPA) mechanisms in SiN at telecom wavelengths [39–41] which provides more stable operation. Yet, while SiN PhC cavities offer the aforementioned advantages, the lower refractive index of SiN compared to silicon renders the quest for high Q-factors more challenging, as the refractive index contrast of the former tends to be lower (∼0.55 in the case of oxide clad structures) [42–45]. The desire to maximize the refractive index contrast to achieve the highest Q-factors has driven the realization of SiN 1D PhC cavity suspended in air (air-cladded), reaching measured Q-factor values up to 10^5^ [46–48]. Despite their higher optical performances, suspended 1D PhC cavities are characterized by two great drawbacks: more complex fabrication and structural/mechanical fragility. To address these issues, great research focus has recently been directed towards the development of new encapsulated high-Q SiN 1D PhC cavity designs, with the best exhibiting a measured Q-factor of 4.42 ⋅ 10^5^ [49]. However, the Q-factor of this type of structures decays very rapidly in asymmetric architectures (where the under-cladding material differs from the upper-cladding), as in the case of SiN 1D PhC cavities lying on the buried oxide (BOx) of a silicon wafer, clad by air or a liquid – necessary to detect the analytes.

In this work, we demonstrate a novel asymmetrically clad 1D PhC cavity design based on a tapered array of SiN rectangular pillars, hereafter referred to as “nanosticks”, in which both the stick shape and sidewalls angles are engineered to achieve intrinsic Q-factors in the 10^6^ ÷ 10^8^ range over a wide set of upper-cladding refractive indexes (from 1.00 to 1.45), with measured Q-factors, that match the engineered sidewalls trends, in the range of 10^5^ (limited by fabrication imperfections). Some of these designs reach record Q-factors of 0.7·10^8^, the highest calculated Q-factor for non-suspended SiN PhC cavities in literature, with measured Q-factors in range of 10^5^ (the highest Q-factor ever measured in asymmetric SiN 1D PhC cavities). In this work, we also show a study on sensitivity of the devices for refractive index sensing, and we propose the concept of a refractive index sensor based on a hybrid external cavity laser (HECL) employing the SiN asymmetric nanosticks as wavelength selective (resonant) mirrors. Finally, we demonstrate mode-hop free single mode lasing operation of the SiN nanostick based HECL in the telecom range, achieving power outputs in the range of mWs and side mode suppression rate (SMSR) of ∼40 dB, comparable to the performances of SiN DBR lasers [50] in telecom applications, while reducing the footprint by more than an order of magnitude. The HECL device is also shown in operation as a refractive index (RI) sensor by measuring its lasing wavelength shift in the presence of air or de-ionized water as upper-cladding. This is the first demonstration of a HECL-based RI sensor, which avoids the external off-chip light source required by MRRs passive RI sensors, with the further advantage of the 1D PhC cavity HECL having a reduced complexity and footprint compared to MRR based HECLs.

Avoiding the rapid drop in optical performances of asymmetric architectures over a broad range of upper-cladding refractive indexes, allows the employment of these devices in HECL configurations to detect low concentrations of analytes both in air and a wide range of solutions. This, coupled to the SiN biocompatibility and transparency from the visible to the NIR spectrum, paves the way to the development of a new set of chip-integrated, highly sensitive and mass manufactured label-free optical sensors for air and liquid environment.

## 2 Cavity concept and design

A large number of 1D PhC cavity designs exhibiting ultrahigh Q/V have been demonstrated for silicon [38, 51, 52], in which hole size, periodicity, and shape are used to achieve strong optical confinement in the cavity. The SiN cavity design used in our work is of the stack mode-gap type consisting in a periodic array of SiN rectangular sticks divided by air (or any other upper cladding material) gaps, acting as wavelength selective mirrors as in high contrast Bragg gratings, lying on silica.

**Figure 1:**
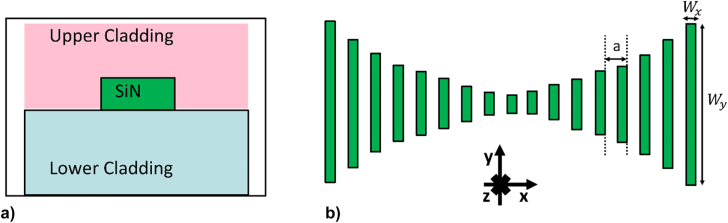
Schematics of the 1D PhC, showing its tapering law: (a) Cross-section of the structure, (b) quadratic tapering of the sticks of the cavity.

The nanostick cavity is generated by parabolically tapering the width of the sticks, which effectively creates a defect in the photonic bandgap (PBG) and induces a Gaussian-shaped electric field distribution along the cavity that minimizes the optical losses [53], since the smooth perturbation increases the mirror strength [54]. For the study on the Q-factor of the 1D PhC cavity, different tapering laws have been investigated (Figure S.1 in the Supplementary) with the quadratic law, shown in the schematics of Figure 1, leading to Q-factors higher than 10^7^. The quadratic tapering law is, therefore, used for the further investigation of all the other geometry parameters. Indeed, for this work the following tapering function has been chosen for the nanostick cavities:

(1) $$W_{y}\left(i\right)=W_{y}\left(0\right)+\frac{i^{2}}{i_{\max}^{2}}\left[W_{y}\left(i_{\max}\right)-W_{y}\left(0\right)\right]$$

where $W_{y}\left(i\right)$ is the width of the *ith* stick along the *y*-axis, with *i* being the number of the single stick and *i*_max_ the number of the sticks for each side of the cavity (from the central stick with *i* = 0), and $W_{y}\left(0\right)$ is the width of the stick at the centre of the cavity, while $W_{y}\left(i_{\max}\right)$ the width of the last stick at both ends of the PhC, with:

(2) $$W_{y}\left(0\right)=3a$$

(3) $$W_{y}\left(i_{\max}\right)=2\cdot W_{y}\left(0\right)=6a$$

*a* representing the periodicity of the 1D PhC along the *x*-direction, while keeping the stick length *W*_*x*_ constant. The variation of the sticks widths along the *y*-axis offers a much broader range of refractive index modulation compared to the *x*-axis, as the latter is limited by fabrication constraints due to the sub-μm features of the PhC gaps, while a μm scale width variation is possible along the *y*-direction. The 1D PhC cavities considered in this work have been designed for a 300 nm thick SiN platform (with refractive index *n* = 2.00 in the telecom range) on a 2 µm thick layer of BOx (*n* = 1.45). To achieve high Q-factor, the stick length along the *x*-axis, *W*_*x*_, and periodicity, *a*, are studied through finite difference time domain (FDTD) software (Lumerical FDTD solutions, Synopsis RSOFT and MEEP), while always maintaining a *W*_*x*_/*a* ≤ 0.5 so that a sample fluid can effectively cover all the surface of the structure, maximizing its interaction with the optical field.

**Figure 2:**
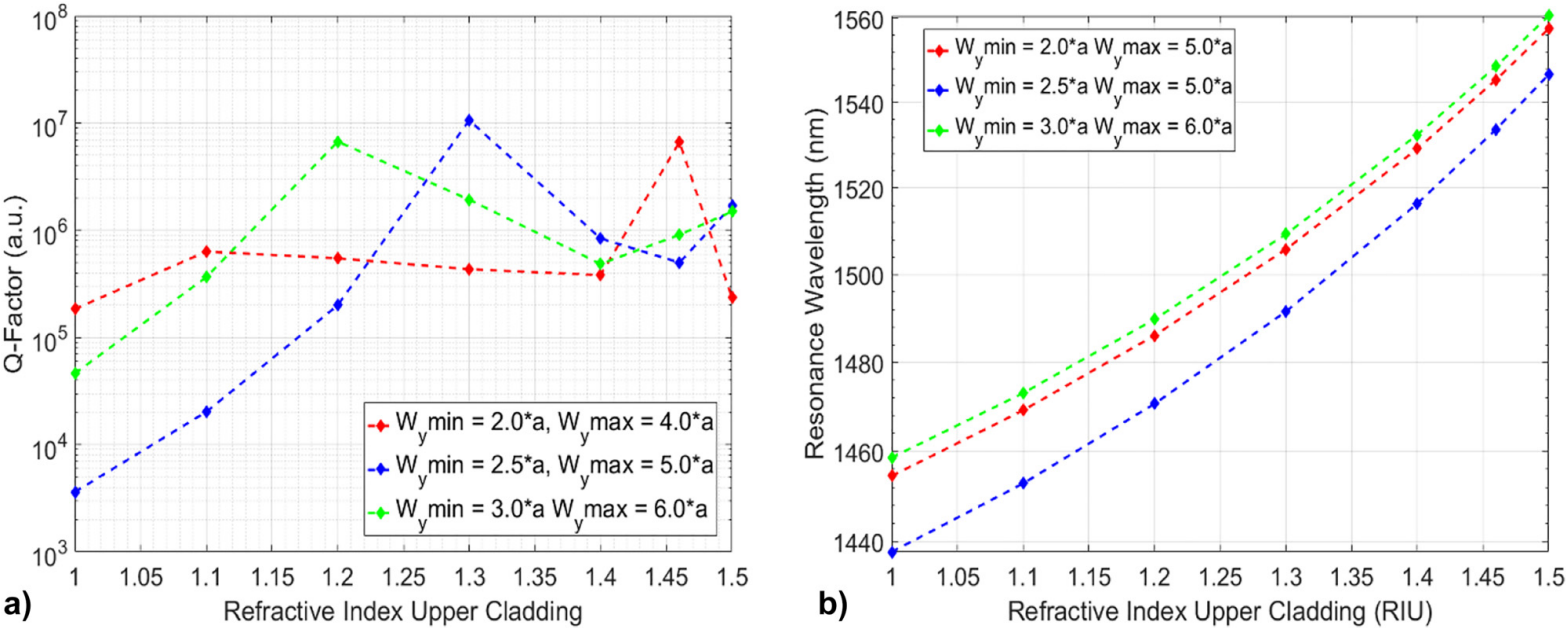
PhC cavity design, (a) Calculated Q-factors with varying upper-cladding refractive index. Each Q-factor curve represents a different quadratic tapering function, which is defined by width of the central and last SiN sticks, *W*_y_min and *W*_y_max, respectively. (b) Calculated resonant wavelength with varying upper-cladding refractive index in the different tapering configurations.

**Figure 3:**
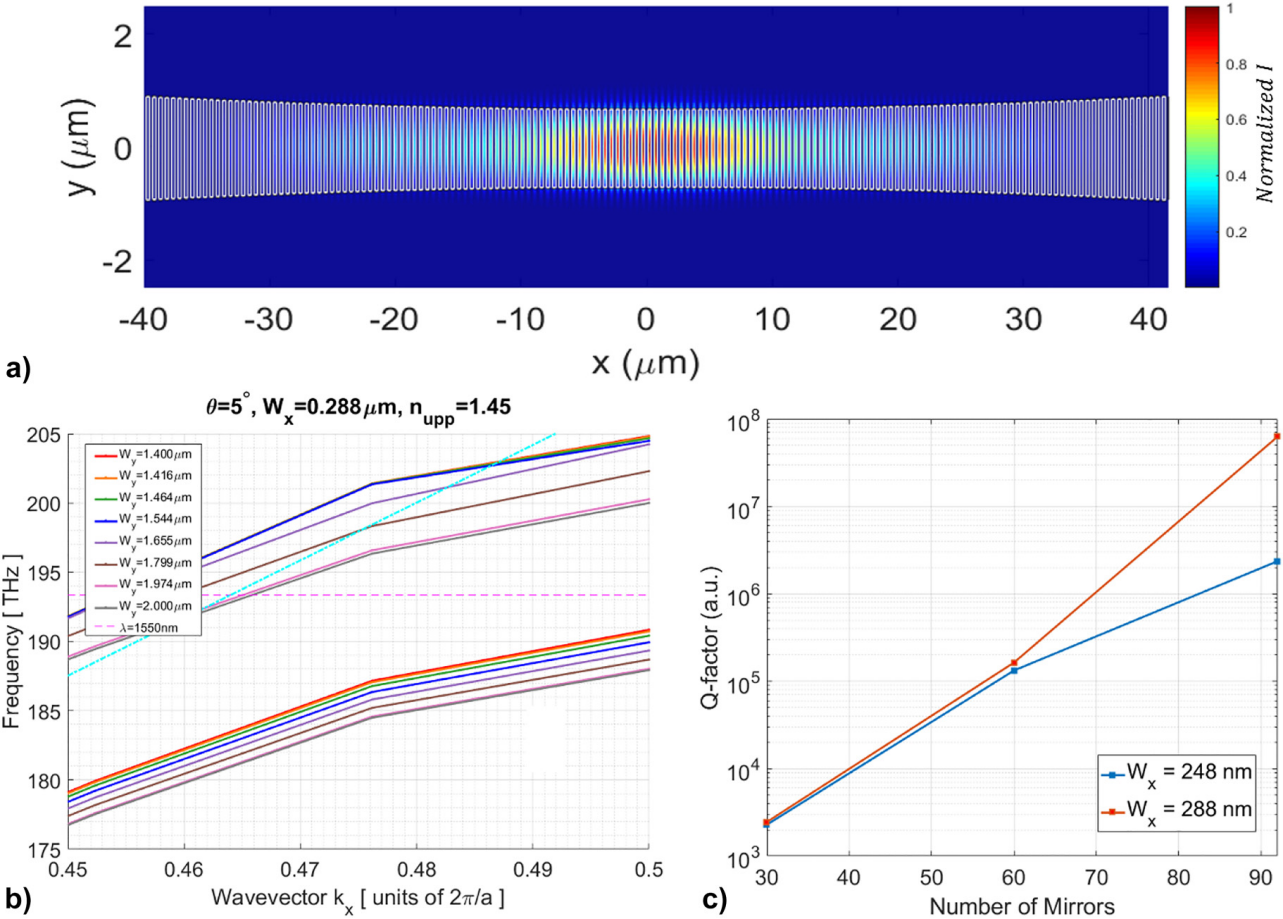
PhC cavity design, (a) Calculated 1D PhC cavity optical resonance for the configuration with one of the tightest optical confinement with silica cladding case and quadratic tapering with normalised intensity, achieving a Q-factor of ∼70 M, (b) photonic band diagram of the silica cladded 1D PhC cavity, turquoise dashed line corresponds to the silica light-lines, (c) Calculated Q-factors against the numbers of mirrors (periods of the 1D PhC).

The Q-factor trends and resonant wavelength shifts with increasing upper-cladding refractive index are shown for different tapering configurations in Figure 2a and b, respectively. By “upper-cladding” we mean the environment on top of the 1D PhC (as shown in Figure 1a), which may be air, liquid or solid. The optical mode confinement in one of the nanostick cavities is shown in Figure 3a (and plotted for other cladding configurations in Figure S.2)**,** which depicts the intensity profile ${\left|E_{y}\right|}^{2}$ , with a calculated Q-factor of ∼70 M Silica-cladded architecture in this case, a discussion on asymmetric cladding is detailed later in this work). In all the configurations, more than 70% of the optical mode electric field is confined over the substrate (as calculated in the Supplementary Section S1). Figure 3b shows the band structures corresponding to increasing values of the stick *W*_*y*_, from 1400 to 2000 nm, highlighting the effect of tapering on the photonic bandgap of cavity (a detailed band structure analysis is shown in the Supplementary S6). Apart from the sticks widths tapering function, the intrinsic Q-factor of the nanostick cavity is dependent on the number of mirrors (sticks) composing the system (Figure 3c) and the interplay between the periodicity of the PhC and the sticks length along the propagation. As shown in Figure 3b, the highest Q-factor is achieved with 90 or more periods at each side of the central stick.

**Figure 4:**
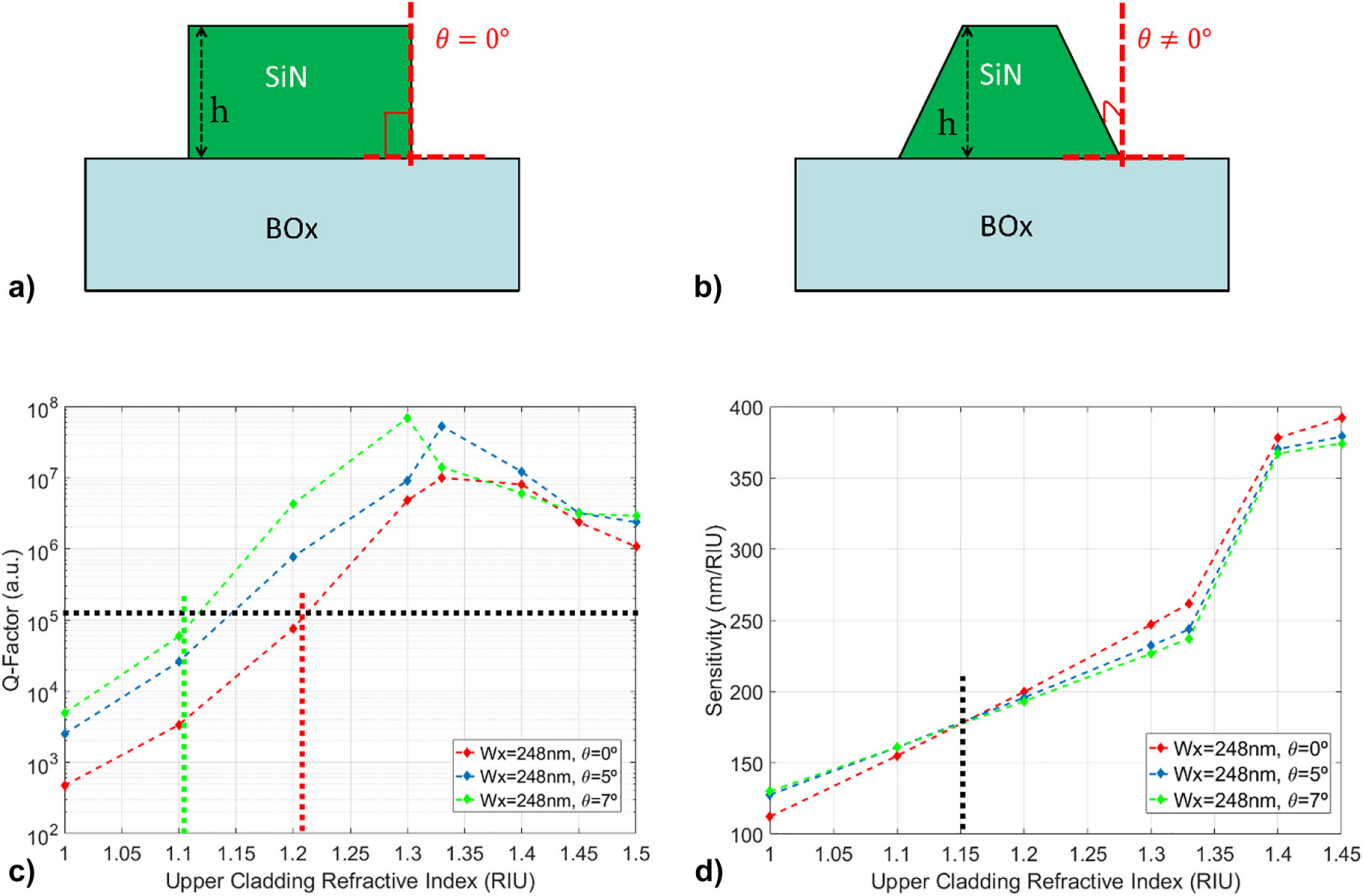
PhC cavity sidewall angle design, (a) and (b) Schematics of the 1D PhC cavity sticks illustrating different sidewall angles. The upper base of the trapezoid has the same size of the rectangular stick base. (c) Calculated Q-factor trends with varying upper-cladding refractive index for constant stick width of 248 nm and different sidewall angle values (0°, 5° and 7°). The red and green dashed lines refer to lowest refractive index with a Q-factor equal to 10^5^ (black dashed line). (d) Sensitivity against upper-cladding refractive index of the 1D PhC cavity for the same sidewall angle values in (c).

For the optimization of the asymmetrically cladded cavity design, a study of the nanostick system response to different upper-cladding refractive indexes and SiN stick sidewalls angles have been performed. This approach has been carried out by means of 3D FDTD simulations of the asymmetric system with the upper-cladding refractive index varying from 1 to 1.5 with steps of 0.1. To engineer the Q-factor of the asymmetric system, the optical response study with different upper-cladding has been performed using the same nanostick cavity geometry, while varying the SiN stick sidewalls angles: 0°, 5° and 7°, respectively. These sidewalls angles values were chosen as they can be achieved by varying the fabrication conditions. A schematic of the nanostick cross-section is shown in Figure 4a and b for the rectangular and trapezoidal cross-sections, respectively. The 1D PhC cavity has been simulated in all upper-cladding and sidewall angle configurations for a wide range of stick widths (*W*_*x*_), showing high optical performances for all the studied range in any configuration (as seen shown in Figure S.3 of the Supplementary), demonstrating the cavity resilience to fabrication imperfections.

Figure 4c shows the calculated Q-factor of the 1D PhC cavity with increasing upper cladding refractive index in the case of different sidewalls angles, while maintaining the same stick width. In this plot, the effect of the sidewall angle on the optical performances of the cavity is manifested by the fact that steeper etching angles are broadening the high Q-factor features of the cavity over wider ranges of upper cladding refractive indexes, from the 1.2 ÷ 1.5 of the *θ* = 0° configuration to the 1.1 ÷ 1.5 of the *θ* = 7° configuration. The curves also show maximum Q-factors of $∼0.7\cdot 10^{8}$ at upper cladding RI of 1.3 and ∼10^7^ at RI of 1.35 for the *θ* = 7° and 0°, respectively. Figure 4d shows the calculated sensitivities, *S*, here defined as Δ*λ*/Δ*n*, for the same cavity configurations in Figure 4c. The plot interestingly shows higher sensitivities for the trapezoidal stick configurations compared to the rectangular one in the most asymmetric cladding systems (from 1 to 1.15 as indicated by the black dashed line), while the trend reverses towards more symmetric cladding systems, in which the higher sensitivities are achieved by the rectangular stick configuration. This fact also underlines the great potential of the trapezoidal cavity configurations for sensing.

**Figure 5:**
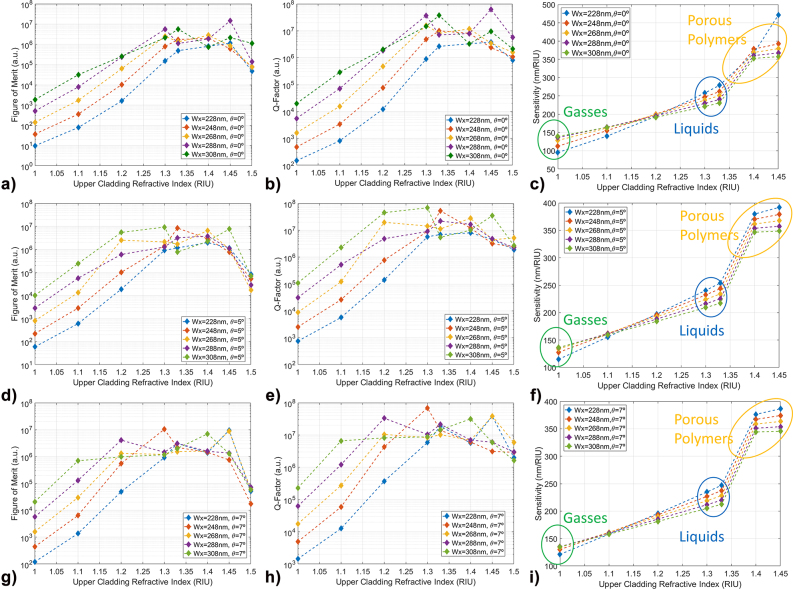
Simulated Figure of merit, Q-factor, and sensitivity trends with increasing upper-cladding refractive index in the different stick width and sidewall angle configurations. Each row of plots corresponds to the same sidewall angle values: (a), (b) and (c) are related to *θ* = 0°; (d), (e) and (f) correspond to *θ* = 5°; (g), (h) and (i) correspond to *θ* = 7°. In each plot, the different coloured curves are related to different sticks width (*W*_*x*_). Coloured ellipses represent area of particular interest for sensing in gasses (green), liquids (blue) and common porous polymers (yellow).

The possibility of employing this type of SiN PhCs as optical sensors has been further investigated by calculating figure of merit (FOM), Q-factor and sensitivity of the various nanostick cavity designs with varying upper-cladding refractive indexes for different cavity stick widths (228, 248, 268, 288 and 308 nm) in each sidewalls angle configuration: 0° in the top row of plots, 5° in the middle row and 7° in the bottom row of plots in the panel of Figure 5, with the FOM being defined as:

(4) $$FOM=\frac{S}{\delta \lambda }=S\cdot \frac{Q}{\lambda_{\text{res}}}.$$

where *S* represents the sensitivity, *Q* the Q-factor and *λ*_res_ the wavelength of the nanocavity selected optical mode. In Figure 5a and b, Figure 5d and e and Figure 5g and h is evident the shift of the FOM and Q-factor bell-shaped curves towards lower upper-cladding refractive indexes with increasing stick widths (*W*_*x*_) in each sidewall angle configuration, enabling the possibility to tune the performances, range of operation and operating environment of the 1D PhC cavity through the *W*_*x*_ parameter, which can be lithographically controlled to the nanometre with relative ease. Figure 5c, f, and i depict the sensitivity against upper-cladding RI for the stick width parameter sweep in the three different sidewall angle configurations (0°, 5° and 7° respectively), showing sensitivity values in the range of hundreds of nm/RUI over all the refractive range considered, with particular focus in the RI ranges around 1 for sensing with gasses as upper-claddings, near 1.33 for liquids and over 1.33 for porous polymers (e.g., SU8). While all geometries exhibit high performance over the upper-cladding RI space simulated, the configurations with *W*_*x*_ = 308 nm and 7°, *W*_*x*_ = 248 nm and 7° and *W*_*x*_ = 228 nm and 0° are optimal for upper-cladding RI values of 1 (air, gases), 1.3 (liquids) and 1.4 (spin-coated polymers), respectively. The detection limit associated to the simulated results, defined in Eq. (8) in Section S.4 of the Supplementary (derived from [55]), can be calculated over all the investigated upper-cladding RI range. From the Q-factors and sensitivities shown in Figure 5, detection limits as low as of 4.6 ⋅ 10^−5^ RIU and 1.1 ⋅ 10^−7^ RIU are calculated for upper-cladding RI near 1 (gases) and 1.3 (liquids) respectively (for *W*_*x*_ of 308 nm and sidewall angles of 5° and 7°). The asymmetry between the top and the bottom cladding causes coupling between TE like and TM like modes. We believe that the sidewall angle changes the overlap of the TE mode with the SiN, reducing the TE-TM mode conversion.

So far, the isolated cavity configuration has been discussed, while an important part of any integrated optical sensor device is the injection and detection of light into and from the cavity. The accessing of the 1D PhC cavity through a strip waveguide has been investigated in many configurations, to evaluate the better solution in terms of optical performances and ease of fabrication and characterization (Figures S.5 and S.6 in the Supplementary Material [56–58]). The chosen coupling approach, referred to as side-coupling, comprises the implementation of a SiN strip waveguide on the side of the PhC cavity, whose guided mode interacts evanescently with the latter. This coupling scheme only requires one lithographic step, easing the fabrication of the integrated waveguide-cavity system. The waveguide-microcavity side-coupling approach has also been investigated and optimized, as reported in Figure S.5 of the Supplementary Material.

## 3 Fabrication and measurements

The integrated waveguide-cavity systems, discussed in Section 2 **,** have been fabricated through conventional nanofabrication processes in which the processing steps were adjusted in order to achieve the required sidewall profiles. For this work, PhC cavities designed to be resonant in the telecom wavelength range from 1500 nm to 1600 nm have been fabricated.

**Figure 6:**
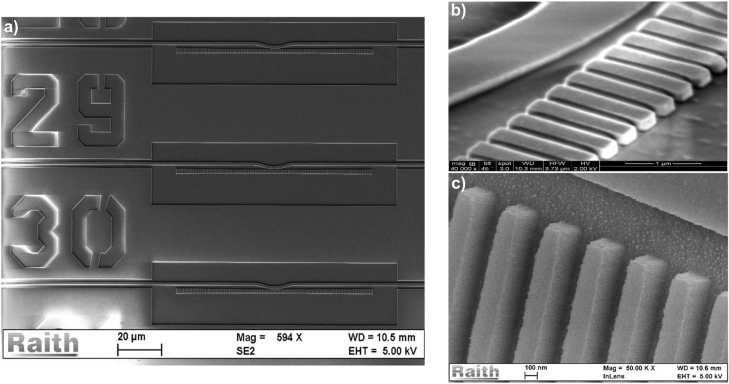
SEM pictures of the fabricated devices: (a) SEM image of the quadratically tapered devices taken normal to the chip surface, (b) SEM image of the rectangular cross-section devices taken at a 45° angle and (c) SEM image of the trapezoidal cross-section devices taken at a 45° and measuring an etching angle of ∼7°.

First, a 450 nm thick layer of ZEP 520A resist was spin-coated on a 300 nm thick layer of SiN deposited with plasma enhanced chemical vapour deposition (PECVD) on a thermally oxidised 4” bulk Silicon wafer (BOx thickness of 2 µm). Then, the devices layouts have been patterned on the resist by means of electron beam lithography (EBL) using 100 kV voltage and later developed in a bath of n-Amyl Acetate solution for 90s and rinsed with IPA. The patterns were then transferred to the PECVD SiN layer through inductively coupled plasma (ICP) etch step in SF_6_:CHF_3_ chemistry in a 3:5 ratio (etch rate of ∼45 nm/min). The residual resist was later removed through an O_2_ plasma ashing step and a bath in 1165 Remover for 30 min. The different stick sidewalls profiles have been controlled by varying the ICP power and etch pressure, achieving etching angles (*θ*) of 0° and 7°, with etch pressures of 2.5 mTorr and 10 mTorr respectively (Figure 6). Lastly, one of the chips etched with 2.5 mTorr of pressure and one of the ones etched with 10 mTorr where spin-coated with spin-on-glass (SOG – Accuglass, RI ∼1.4) to obtain 4 different sidewall angle – upper-cladding configurations.

**Figure 7:**
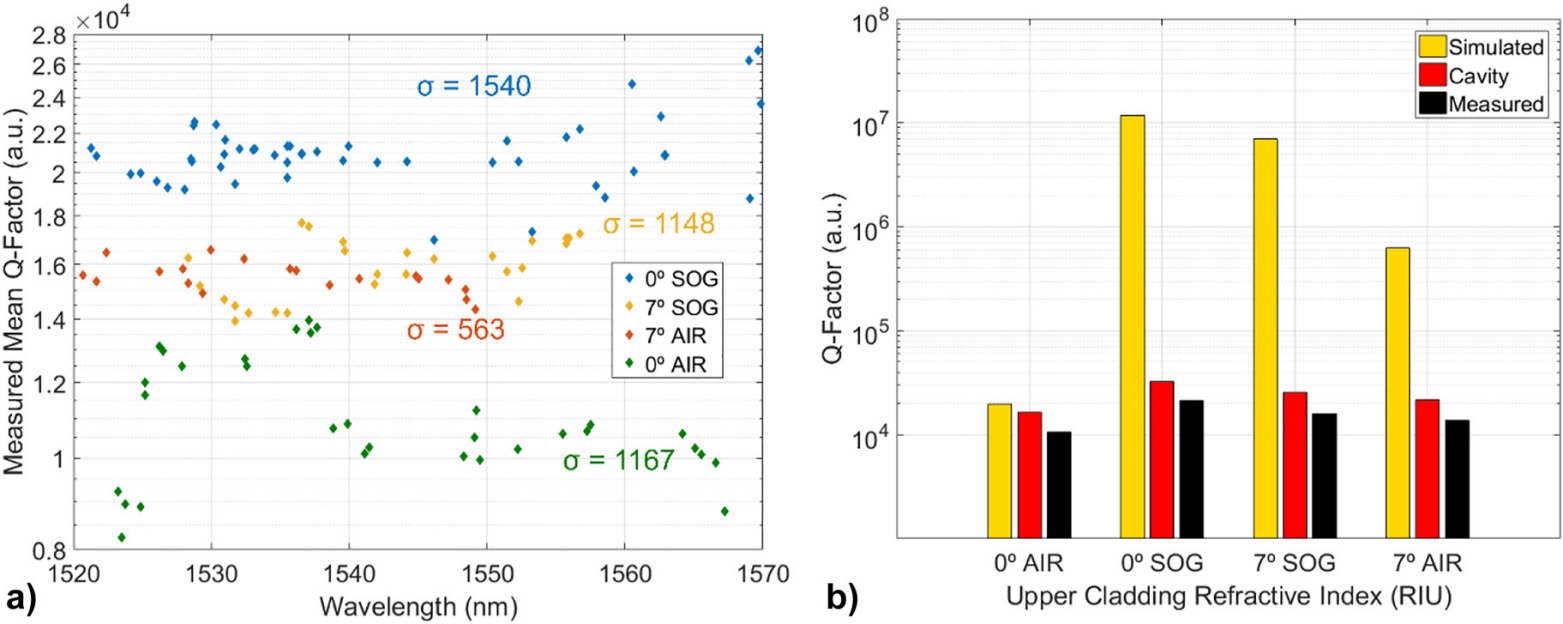
Experimentally measured PhC cavities, (a) Measured average Q-factor against wavelength of all the measured devices, different colours correspond to different upper-cladding and etching angle configurations: 0° – air (green), 7° – air (red), 0° – SOG (blue) and 7° – SOG (yellow). Each data point is an average of 3 devices with the same configuration operating at that wavelength (b) plot of averaged Q-factors over all the devices in the different configurations on the *x*-axis. The yellow bars correspond to calculated Q-factors, the red bars are associated to cavity Q_0_ and the black bars to measured-factors, Q_meas_.

Following fabrication, the different devices have been firstly optically measured by injecting the light emitted by an ASE broadband source (Amonics ALS) into the waveguide through a system of fibres and spot-size converting lenses, collecting the output of the waveguide containing the cavity spectral response into a photodetector (similar to the setup shown in [59]). A summary of the measured spectra and lithographic tuning (sweeping the *W*_*x*_, *a*, side-coupling gap and waveguide coupling shape parameters, while maintaining $W_{y}\left(0\right)$ = 1.4 µm and $W_{y}\left(N\right)$ = 2.0 µm) is reported in Figures S.10 and S.11 in the Supplementary Material, which confirm the simulated trends and side-coupling behaviour of the waveguide-microcavity system. The Q-factors of all the fabricated devices in all the four sidewall profiles – upper-cladding configurations have been measured by fitting each optical resonance with a Lorentzian, and the overall results are shown in Figure 7, with Figure 7a depicting the average Q-factor of the devices for each configuration, in which each data point is an average of 3 devices operating at the same wavelength, highlighting the optical performance trends for each etching angle-upper cladding combination, and Figure 7b showing the plot of the Q-factors averaged over the same device sidewalls angle – upper-cladding configurations compared to the Q-factors predicted by the FDTD simulation for the same configuration, for which the best configuration is the 0°-SOG, followed by 7°-SOG, then 7°-AIR and lastly 0°-AIR (Figure 4c).

As Figure 7a shows, the SOG-cladded group etched at 0° was the one showing the greatest number of better performing devices, with measured Q-factors in the range $\left(1.0-9.0\right)\cdot 10^{4}$ , as expected from the calculations. To compare the optical performances in the various upper-cladding and sidewall profile configurations with the predicted behaviour, the measured Q-factors have been averaged over all the devices in each of the four categories, obtaining a general overview on the performances of each $\left(n_{\text{upp}},\theta \right)$ set. Figure 7b depicts the highest simulated Q-factors for each investigated angle-cladding configuration among the fabricated geometries, superimposed to the measured Q-factors (averaged over all the measured devices geometries on each single chip) corresponding to each angle-cladding chip configuration. The results summarized in Figure 7b verify the predicted trends (shown in Figure 4c), with the best performances achieved by the $\left(n_{\text{upp}}=1.40,\theta =0{}^{\circ}\right)$ group, followed in sequence by the $\left(n_{\text{upp}}=1.40,\theta =7{}^{\circ}\right)$ , the $\left(n_{\text{upp}}=1.00,\theta =7{}^{\circ}\right)$ and the $\left(n_{\text{upp}}=1.00,\theta =0{}^{\circ}\right)$ groups.

**Figure 8:**
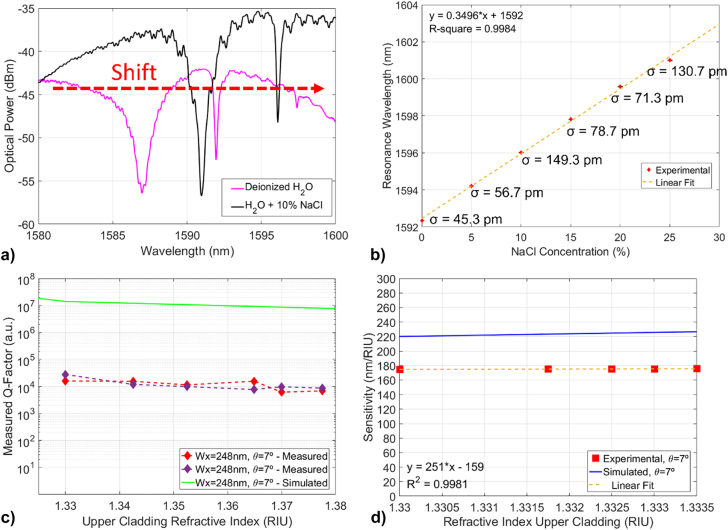
PhC cavity sensing measurements, (a) Optical spectra of a nanobeam device with the presence of de-ionised water (magenta) and [10%] NaCl solution (black) on top, with the red dashed line indicating the resonance shift with the different upper cladding RI, (b) Shift of the resonance wavelength of the microcavity with salt concentration, plotted with error bars and standard deviations, (c) measured Q-factors of the microcavity resonances with upper cladding RI, compared to the simulated Q-factor (green) for the same device architecture and (d) measured sensitivity of the microcavity against increasing upper cladding RI (experimental datapoints taken in the region of RI from 1.3300 to 1.3335).

The working principle of the 1D PhC cavity refractive index sensors has been tested by measuring the optical response of the air-cladded devices (*θ* = 7°) with the presence of either de-ionised water or a 10% NaCl aqueous solution, deposited onto the sample by means of drop-cast with a µl pipette. For this measurement, the sample was maintained at constant room temperature *T*_room_ = 20.1 °C, through a Peltier element mounted on the aluminium sample-holder. The spectra collected after the different solution drop-casts (and associated rinse and dry cycle), shown in Figure 8a, exhibit an abrupt wavelength shift of the resonant modes wavelength towards the red while maintaining the high Q-factor of the fundamental modes, as expected for this device architecture.

**Figure 9:**
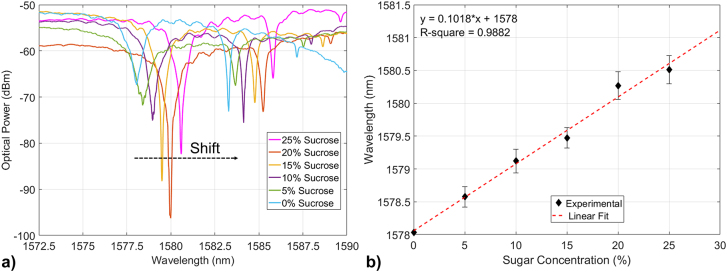
PhC cavity wavelength shift with solution, (a) Measured optical spectrum of the microcavity for different sugar concentrations (from 0% to 5, 10, 15, 20 and 25%) and (b) plot of the microcavity resonance wavelength against sugar concentration, in which the red-dashed line represents a linear fitting of the datapoints. The standard deviations related to these measurements are shown in Figure S.14 of the Supplementary Materials.

The spectra of the 1D PhC cavities have been measured after a drop-cast of NaCl solutions at different concentrations, with an acetone and de-ionised water rinse and dry cycle between every drop-cast. The shift of the device resonance wavelength is plotted against the increasing concentration of NaCl (from 0% to 25% in steps of 5%) in Figure 8b (where the corresponding refractive index of the solution has been calculated according to [60] and plotted in Figure S.8 in the Supplementary Material), exhibiting a clear linear behaviour. Moreover, the measured Q-factors of the resonances shifting with the solution RI is instead plotted in Figure 8c, depicting a good match with the simulated trend. Measurements with the previously discussed modalities have been repeated with finer NaCl concentration steps (0.00, 0.25, 0.50, 0.75 and 1.00%), with the associated measured sensitivity plotted in Figure 8d, showing a trend similar to the calculations of the corresponding cavity design. We attribute the small mismatch between the experimental and predicted values in Figure 8d to incomplete filling of the voids by the liquid. The detection limit associated to this microcavity is ∼1.7 · 10^−3^ RIU, using [55]. Similar measurements have been performed using solutions of increasing concentration of sucrose (C_12_H_22_O_11_ – the most common form of sugar) dissolved in de-ionised water. The microcavity resonance wavelength shift with the increasing sugar concentration is shown in Figure 9, exhibiting linear behaviour.

The collected microcavity optical spectra with different sugar solutions have been plotted in Figure 9a, where the resonance wavelength red-shifts with increasing sugar concentrations (from 0% to 5, 10, 15, 20 and 25%). The microcavity used for these measurements is one with sticks width (*W*_*x*_) of 248 nm and 7° of sidewall angle. Interestingly, also the resonance measured Q-factor increases from ∼10^4^ to ∼10^5^ with increasing solution RI (from 1.33 of DIW to over 1.355 for the 15, 20 and 25% sugar solutions), similar to the trend observed in the simulations for the same microcavity geometry (Figure 5h). The RI values corresponding to the different sugar concentrations and measured sensitivity are plotted in S.15 of the Supplementary Materials. The sensitivity measured for this microcavity is ∼150 nm/RIU, leading to an experimental detection limit of 1.1 ⋅ 10^−3^ RIU (Eq. (8) in Section S.4 of the Supplementary).

**Figure 10:**
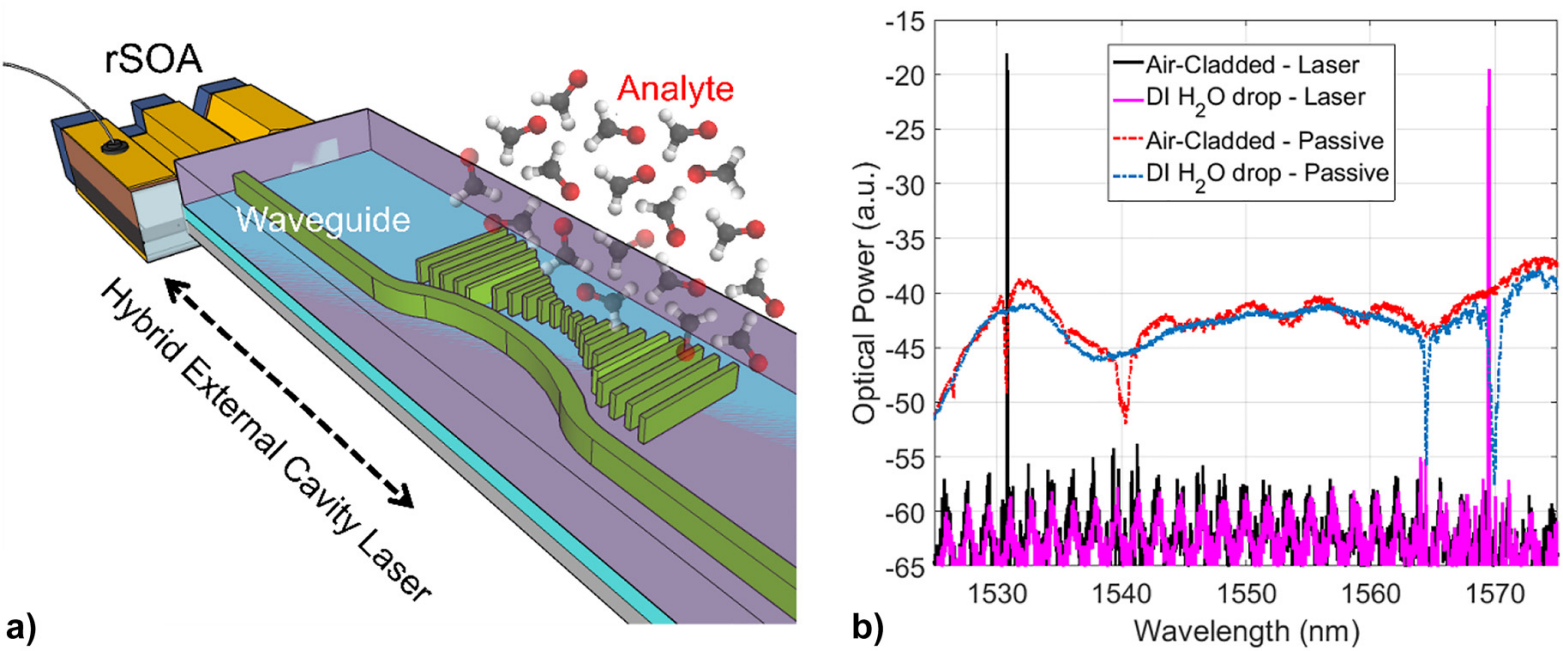
HECL sensor, (a) Schematics of a hybrid external cavity laser (HECL) based on the designed 1D PhC cavity, operating as biosensor and (b) same HECL device lasing with different upper-cladding solutions, in which the lasing wavelength with different solutions is overlapped to the transmission dip of the 1D PhC cavity taken in the same cladding conditions (air in black and de-ionised water in magenta). The lasing wavelength shifts with the shifting of the PhC cavity resonance, as the solution covering the microcavity changes (from air to de-ionised water) solution.

After the characterisation of the optical performances of the nanocavities, a laser based refractive index sensor concept based on these devices is shown in Figure 10. The sensor comprises a commercially available reflective semiconductor optical amplifier (rSOA) butt-coupled to the waveguide connected to the PhC cavity on the SiN chip (Figure 10a), in hybrid external cavity laser (HECL) configuration similar to [50]. The lasing characteristics of this SiN 1D PhC cavity based HECL are measured and reported in Figure S.16 in the Supplementary Material, showing output power in the mW range and hopping-free single-mode lasing regime over a wide range of injected currents (from 25 to 65 mA) and 40 dB of SMSR. In this configuration, a laser line is generated by the overlap between the gain ripple, the reflection peak of the PhC cavity resonance and the laser cavity longitudinal modes. The 1D PhC cavity spectral response is engineered to be dependent on the device upper-cladding (Figure 4c and d **)**, which is effectively related to the environment conditions of the device: the upper-cladding refractive index of the cavity is proportionally affected by the concentration of analytes dissolved in the liquid or gas solution incorporating the device surface. Due to this, the presence of analytes with a certain concentration in the upper-cladding solution shifts the 1D PhC cavity resonance wavelength by a proportional amount, which translates into a measurable shift of the lasing wavelength of the HECL (a schematics of the laser operation is depicted in Figure S.10 in the Supplementary Material **)**. This is shown in the plots of Figure 10b, in which one of the 1D PhC cavities (*W*_*x*_ of 248 nm, *a* of 476 nm and 250 nm of side-coupling gap with the curved waveguide and 7° of sidewall angle) has been measured in HECL configuration in the presence of air as upper-cladding and in the presence of de-ionised water (DIW). The change in the solution from air to DIW covering the device (i.e., the change in the upper-cladding RI) red-shifts the resonance wavelength of the 1D PhC cavity, which in turn controls the lasing wavelength of the HECL.

**Figure 11:**
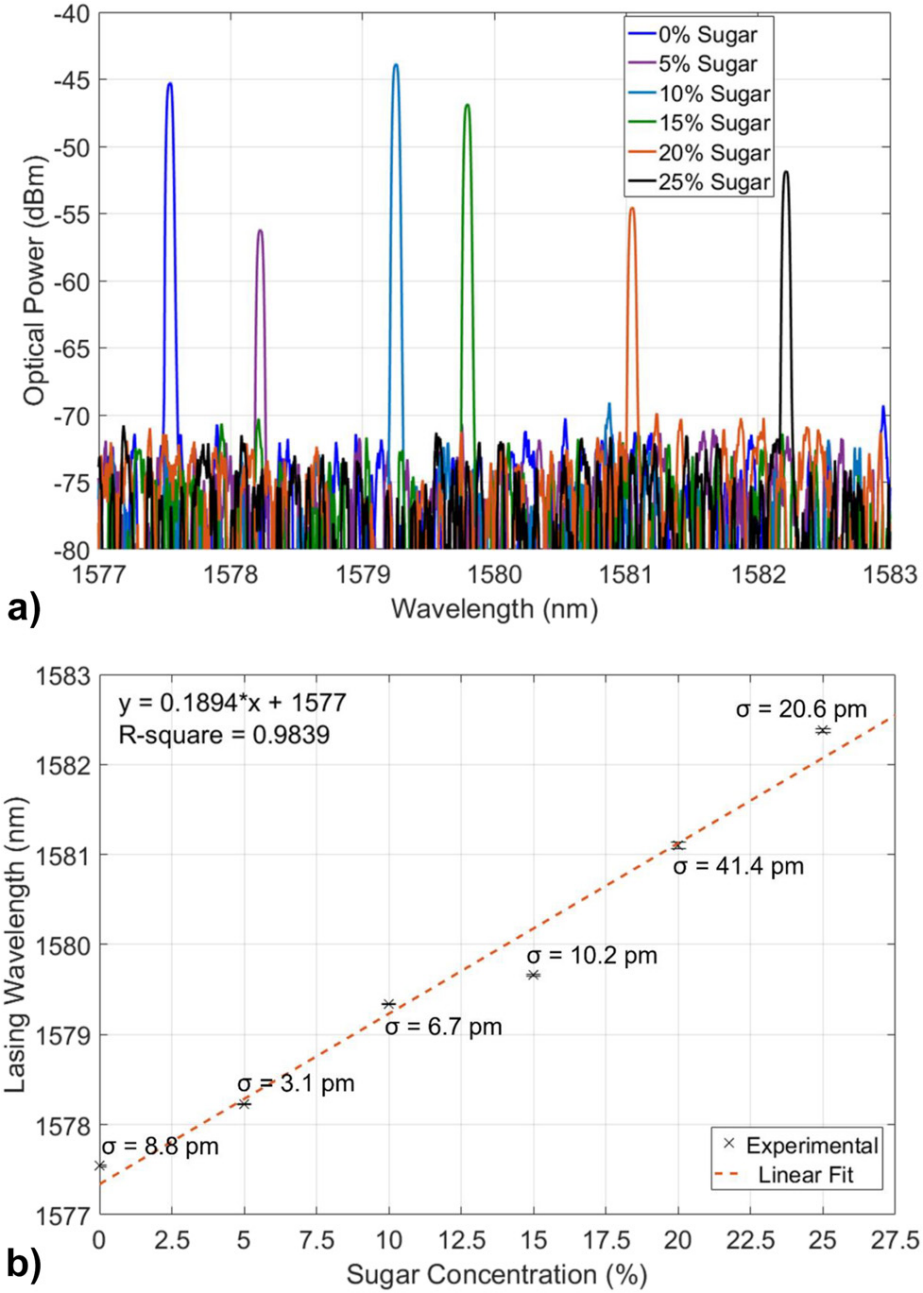
HECL wavelength shift with solutions, (a) Experimental lasing spectra of the HECL based RI sensor in solutions of DI water and increasing concentrations of sugar (C_12_H_22_O_11_), from 0% to 25% and (b) experimental lasing wavelength shift with increasing sugar concentration with error bars and standard deviation, fitted linearly (red dashed line).

The HECL operating as an RI sensor has also been measured in the presence of solutions of sugar in de-ionised water with increasing concentrations, from 0% to 25% in increasing steps of 5%. The corresponding spectra and lasing wavelength shifts are shown in Figure 11, exhibiting approximately linear behaviour with the different sugar concentrations. The standard deviation of the repeated laser measurements at each sugar solution concentration is reported in the plot of Figure 11b. A more detail data analysis regarding these measurements is shown in Section S.5 of the Supplementary Materials. The experimental detection limit associated with the HECL RI sensor is lower than 6.6 · 10^−5^ RIU (value limited by the resolution of the optical spectrum analyser). We believe this to be one of the first demonstrations of the use of a HECL for RI sensing, being characterised by 2 orders of magnitude lower detection limits compared to their passive optical resonator counterparts and compactness and high power (mW range) compared to MRRs-based RI sensors, which also require an external light source.

## 4 Application scenarios of the developed cavities for trace analysis

The developed 1D PhC cavities are versatile building blocks for designing novel sensors for trace analysis in gases, liquids and suspensions. As demonstrated in this paper, they can be employed for reading out changes in the refractive index which are induced by the analyte under investigation, e.g., by measuring the shift in wavelength when the 1D PhC is used in a hybrid EC-laser. The refractive index of a liquid is a volume independent quantity [61]. Therefore, refractive index detectors are highly competitive in terms of sensitivity with volume dependent detectors, such as mass spectrometry or absorbance and fluorescence-based spectroscopy, especially in miniaturized lab-on-a-chip systems. We see great application potential of the 1D PhC cavities as label free detectors in nanoscale separation systems such as nano-HPLC or capillary electrophoresis [61]. Due to their small, active area these highly sensitive cavities might also be beneficial to detect individual nanoparticles suspended in liquids. The 1D PhC cavity could also be used as a sensitive transducer in photothermal sensing schemes for gases and liquids. In photothermal spectroscopy, modulated light absorption by the analyte under investigation is performed to induce a periodic modulation of the sample’s temperature and thus of its refractive index [62]. Due to the recent progress in mid-IR laser sources and due to the fact that in photothermal sensing the generated analyte signal is direct proportional to the power of the excitation source and can also be maximized by focussing the light into a small sample volume, this sensing scheme holds special promise for realizing miniaturized and highly sensitive sensors. The use of narrow linewidth mid-IR lasers allows to selectively excite target gas analytes also in complex matrixes and thus to achieve highly selective measurements. This has been recently demonstrated by interferometric cavity assisted photothermal spectroscopy (ICAPS, [63]), which is based on detecting changes in the transmission profile of Fabry–Perót cavities as induced by refractive index resulting from modulated analyte absorption. This detection principle can be miniaturized and made even more sensitive by replacing low Q-factor FP cavities with 1D PhC cavities having significantly larger Q-factors. With respect to liquid sensing a similar approach can be envisioned when using broadly tuneable mid-IR lasers as an excitation source.

## 5 Conclusions

In this work, we demonstrated novel SiN 1D PhC cavity designs based on quadratically tapered sticks, able to achieve Q-factors over 7 · 10^8^ with different cladding configurations. The possibility to tailor the optical performances of the cavities for different operating environments by engineering the etching profile of the sticks is also discussed. The optimization of the Q-factor with different upper cladding configurations is achieved by optimizing the cavity sticks sidewall angle, obtaining very high optical performances (Q-factors over 10^5^) also in the case of asymmetrically cladded cavities, that is with upper claddings refractive index spanning from 1 to 1.45 – reproducing the case of the nanobeam submerged in different gaseous or liquid solutions. The engineering of the optical response of such 1D PhC cavities with the upper-cladding shown in this work enables the possibility of employment of these devices in label-free optical sensing both in liquids and gasses, as demonstrated with NaCl and C_12_H_*22*_O_11_ solutions. Finally, a sensor device concept based on a Hybrid External Cavity Laser (HECL), using these 1D PhC cavities as resonant mirrors, is proposed and their lasing operation is demonstrated, achieving stable mode-hop free single mode regime over a wide range of driving currents, power outputs in the range of mWs and SMSRs over 40 dB, with clear lasing wavelength shift with different upper cladding refractive index conditions, achieving a factor of 17 (limited by OSA) lower detection limit compared to its passive microcavity counterpart. This nanostick-based HECLs could be exploited for a wide range of applications not only in optical sensing but also for telecom and datacom applications. We envision the deployment of commercially available reflective semiconductor optical amplifiers (rSOAs) coupled to arrays of these SiN integrated PhC cavities resonant at different wavelengths in HECL configuration to be used as label free, low limit of detection, low footprint and high-power optical sensors in both gaseous (air-cladded devices) and liquid environments (e.g., with integrated microfluidics).

## 6 Data and materials availability

The datasets used and/or analysed during the current study available from the corresponding author on reasonable request.

## Supplementary Material

Supplementary Material Details
