# Erratum to: High-Q asymmetrically cladded silicon nitride 1D photonic crystals cavities and hybrid external cavity lasers for sensing in air and liquids

**DOI:** 10.1515/nanoph-2022-0580

**Published:** 2022-10-05

**Authors:** Simone Iadanza, Jesus Hernan Mendoza-Castro, Taynara Oliveira, Sharon M. Butler, Alessio Tedesco, Giuseppe Giannino, Bernhard Lendl, Marco Grande, Liam O’Faolain

**Affiliations:** Centre for Advanced Photonics & Process Analysis, Munster Technological University and Tyndall National Institute, Rossa Ave, Bishopstown, T12 P928 Cork, Ireland; Politecnico di Bari, Bari, 70126, Bari, Italy; Tyndall National Institute, Cork, Ireland; POLIBA DEI, Bari, Puglia, Italy; Chemical Technologies and Analytics, Vienna University of Technology, Vienna, Austria

After the publication of this article [1], the authors found out that the following acknowledgement was missing:
