# Supplementary material for: High-Q asymmetrically cladded silicon nitride 1D photonic crystals cavities and hybrid external cavity lasers for sensing in air and liquids
Source: Nanophotonics. 2022 Aug 10;11(18):4183–96. doi: 10.1515/nanoph-2022-0245 (PMC9412843; doi:10.1515/nanoph-2022-0245)
Supplement: Supplementary file 1 — Supplementary Material Details [file j_nanoph-2022-0245_suppl.docx]

**Supplementary Material**

# S.1 Confined Optical Mode in the SiN 1D PhC cavity

The 1D PhC cavity tapering geometry has been numerically investigated by optimizing the performances of an untampered high contrast SiN Bragg grating and then applying different tapering laws to its sticks without changing the other geometrical parameters, from linear to quadratic and cubic tapering. The simulated Q-factors are plotted against tapering geometry in **Fig. S1**, showing the highest optical performances for quadratic tapering. This is physically explained by the fact that the optical mode is gently confined in the quadratically tapered cavity due to the smooth Gaussian-like effective refractive index profile along the tapered structure, as opposed to more abrupt refractive index changes between periods of the cavity in the case of the other tapered geometries (from the cubic and linear geometries to the untampered structure characterised by a refractive index step-function at each lattice period). The smoother the refractive index transition between the defect at the centre of the cavity towards the external mirror, the less the scattering of light confined in it.


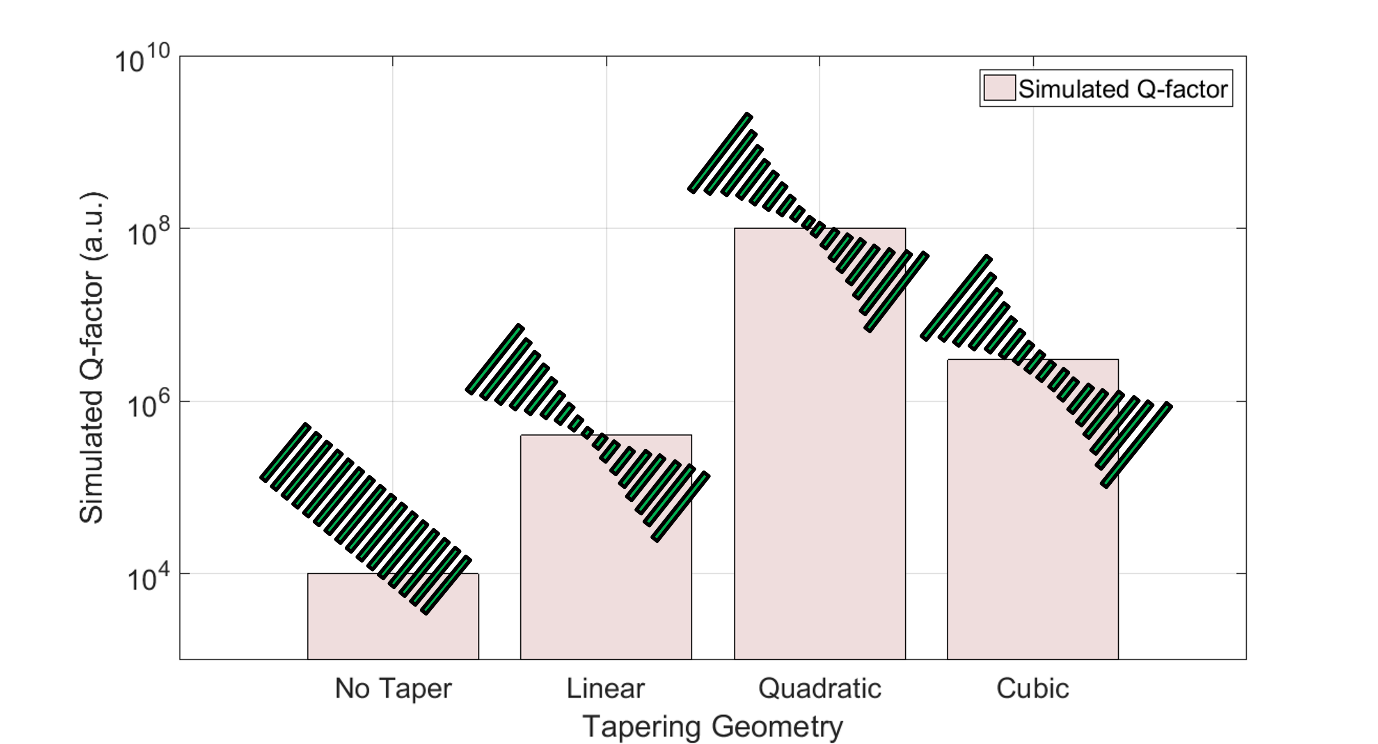


Figure S1 – Simulated cavity Q-factor with different tapering geometries, from untampered high contrast grating to linearly, quadratically and cubically tapered sticks.

The optical modes confined in the 1D PhC cavity have been simulated with an FDTD (Lumerical) software in all the sidewall-angle configurations for many upper-cladding conditions in both xy and xz planes. , **Fig. S.2** shows the optical mode confinement into the cavity in the air-cladded and Silica-cladded configurations, exhibiting in both cases a good portion of the optical energy being distributed in the upper cladding, which further increases the volume of interaction between the resonant modes and the analytes in sensing operation.


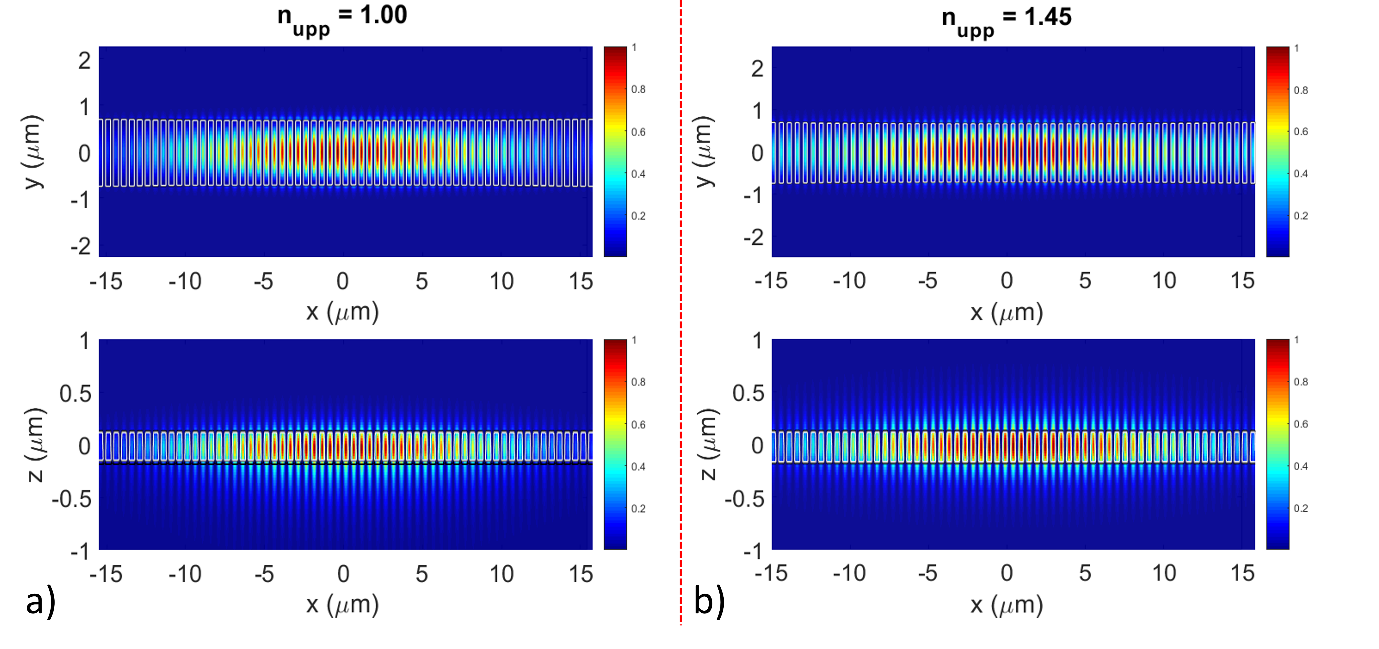


Figure S2 – Calculated optically confined mode in one of the 1D PhC cavity configurations in the xy and xz planes in the case of a) air upper-cladding and b) oxide upper-cladding.

**Fig. S.2** also shows the confined modes expanding in an area of roughly 40 µm^2^ in the xy plane, providing a relatively large sensing area.

The Q-factor trends associated to 1D PhC cavities with increasing SiN stick width (Wx) and etching angles (0º, 5º and 7º) configurations are plotted against increasing upper-cladding refractive index from 1 to 1.5, as shown in **Fig. S.3a**. The Q-factor trends assume the bell-shaped curves reaching record maximum values in the range of 10^8^. The calculated data show a shift of the Q-factor curves peaks towards lower upper-cladding refractive indexes with increasing sidewall angles, leading to the fact that the trapezoidal structures favour the more asymmetric cladding architectures. It is worth nothing that the Q-factor curves broaden and flatten with higher sidewall angles compared to the rectangular stick configurations, offering high Q-factor values ($\geq{10}^{6}$) for a larger range of upper-claddings refractive indexes (as in the case of $W_{x}=308 \mathrm{nm}$ and $\theta=7^{\circ}$ configuration, covering the upper cladding refractive index (RI) range from 1.05 to 1.5), unlocking the possibility to tailor the desired cavity optical performances through the engineering of the SiN stick sidewalls angle.


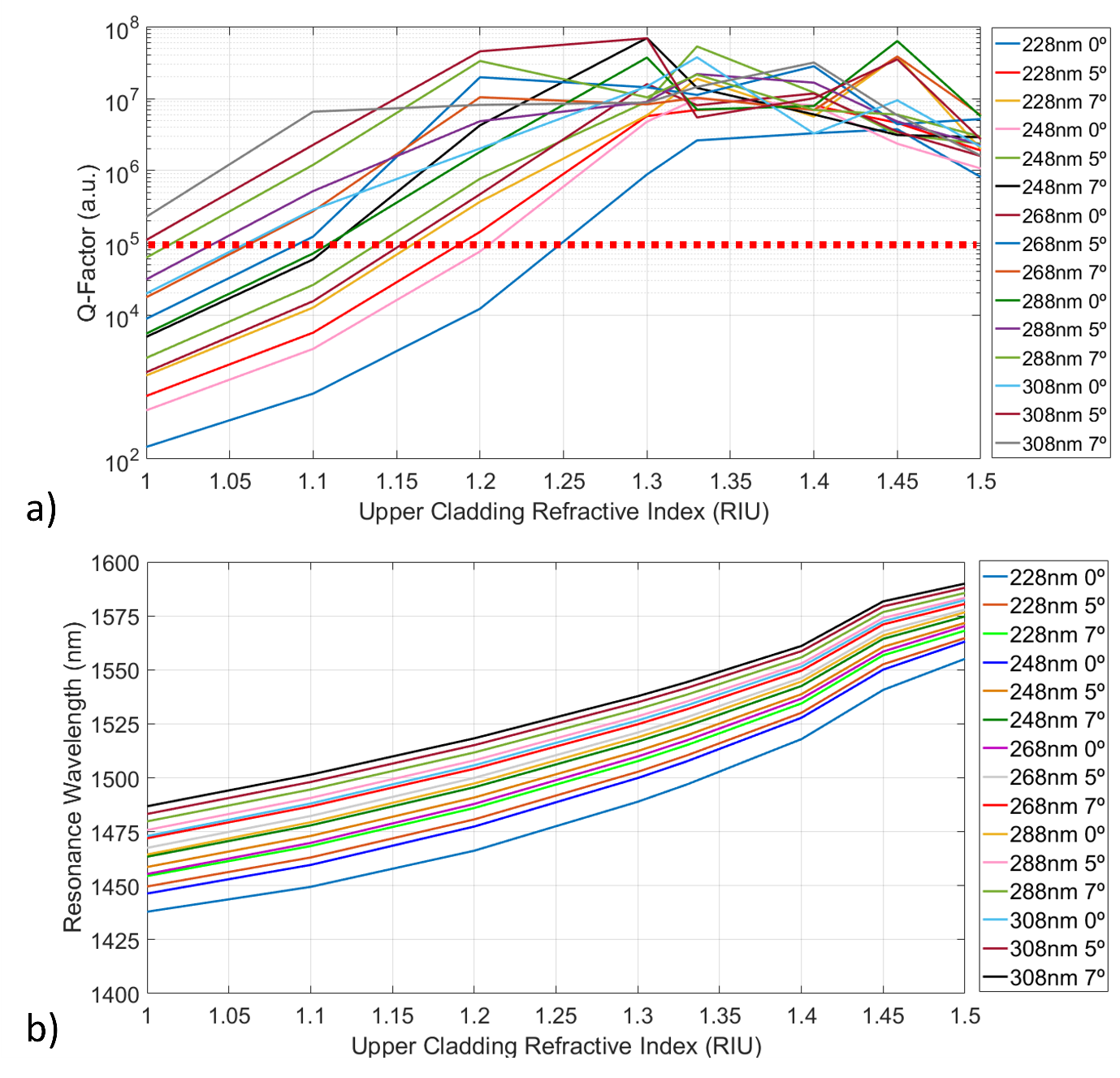


Figure S.3 - a) calculated Q-factor with varying upper cladding refractive index for all the stick width and sidewall angle configurations and b) resonance wavelength shifts with varying upper-cladding refractive index for the same configurations.

Moreover, the main feature of **Fig.** **S.3a** to be highlighted is the fact that there is always a stick-width/etching angle nanobeam configuration which achieves high Q-factors over 10^5^ for every upper cladding refractive index value (from 1 to 1.5), showing that the design has a great resilience against fabrication imperfections and also leading to the possibility to employ these 1D PhC cavities as sensors in any environment, whether gaseous or liquid, choosing among the various designs: e.g. rectangular cross-section cavities for sensing of analytes suspended in high RI diluted polymers and trapezoidal cross-section nanostick cavities for optical sensing in liquids and gasses. **Fig. S.3b** depicts the wavelength of the resonant fundamental mode of each 1D PhC cavity configuration against upper-cladding refractive index, tracking the wavelength red-shift of the resonances with increasing upper cladding refractive index (from 1 to 1.5), spanning hundreds of nanometres.

The optical mode electrical field distribution has also been calculated, to quantify the mode electric field fraction on top of the buried oxide substrate, which acts as sensing volume. This task has been performed by dividing the 3D simulation space into 2D slices in the xz plane and the optical mode fraction calculated through the electrical field of the slice and the following equation [53]:

| $\Gamma_{p}=\frac{\int_{V_{p}} \epsilon\left( \boldsymbol{r} \right)\left\vert\boldsymbol{E}\left( \boldsymbol{r} \right) \right\vert^{2}d^{3}(\boldsymbol{r})}{\int_{V_{T}} \epsilon\left( \boldsymbol{r} \right)\left\vert\boldsymbol{E}\left( \boldsymbol{r} \right) \right\vert^{2}d^{3}(\boldsymbol{r})}$ | (S.1) |
| --- | --- |

Where $\Gamma_{p}$ is the overlap of the mode perturbed by the presence of analytes in the solution, $V_{p}$ the perturbated volume (volume on top of the buried oxide), $V_{T}$ the total volume of the mode, $\epsilon(r)$ the permittivity of the medium and $\boldsymbol{E}(\boldsymbol{r})$ the calculated electric field (in spherical coordinates).


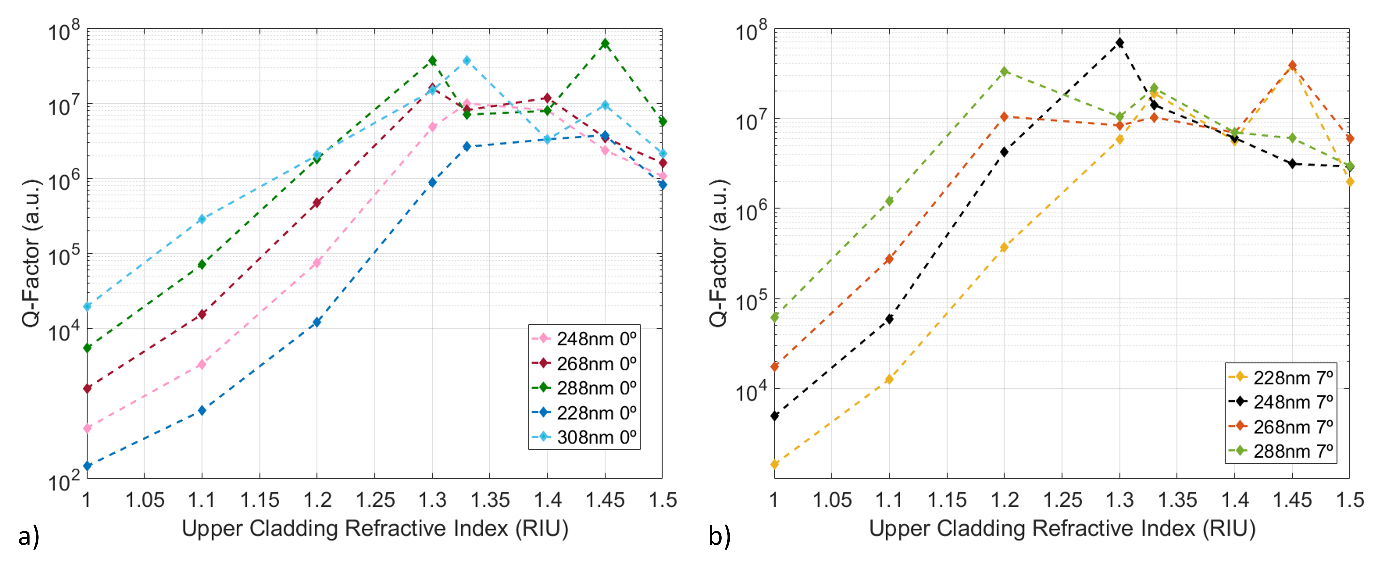


Figure S.4 – Simulated Q-factors of the cavities with increasing upper-cladding RI. Each curve corresponds to a cavity with a different stick width $W_{x}$. a) sidewall angle = 0° and b) sidewall angle = 7°. These also corresponds to the cavity geometries measured.

**Fig. S.4** shows a reduced version of **Fig. S.3a**, separating the curves for cavities with sidewall angle of 0º and the ones with sidewall angle of 7º. These are the cavity configurations discussed in the measurements in section **S.3**.

# S.2 The SiN 1D PhC cavity to a Waveguide

The easiest configuration to inject and extract light from the microcavity comprises the implementation of a SiN waveguide with its light propagation axis parallel to the nanostick (**Fig. S.5a**), as in the case of conventional DBRs. Unlike conventional DBRs, however, the high propagation constant mismatch between the waveguide and the edges of the cavity and the high reflectivity of the latter leads to the incoming light to be totally reflected into the waveguide just after 10 periods (as shown **in Fig. S.5b**), rendering this coupling solution not viable.


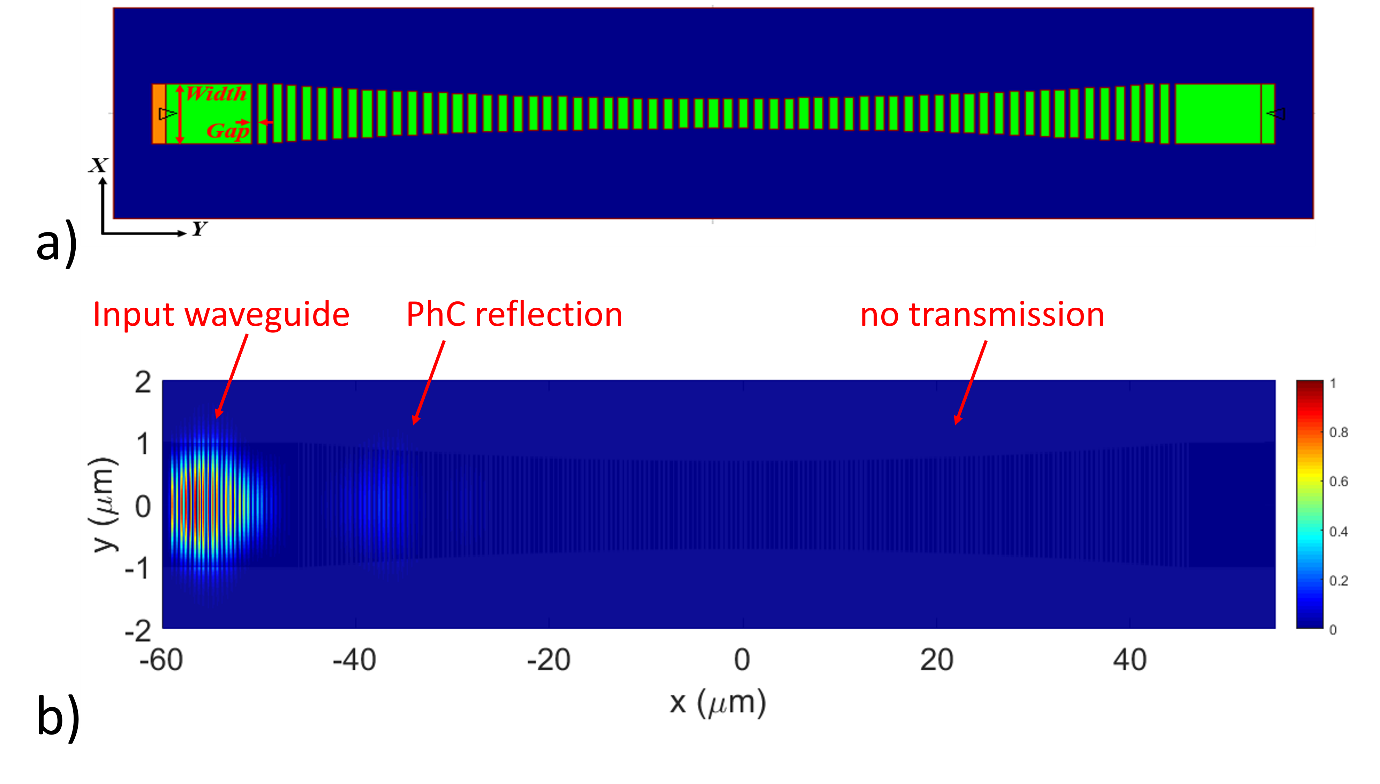


Figure S.5 – a) Schematics of the 1D PhC cavity directly coupled to a waveguide with its axis of light propagation in line with the cavity and b) Simulated propagation of light from the waveguide to the cavity, showing almost all the light reflected back to the input after a few periods of the PhC.

Another waveguide-cavity integration approach consists in the coupling of light vertically from a waveguide to the cavity, as demonstrated with 2D PhC cavities in [57] and [58]. However, this approach requires: the deposition and thickness fine tuning of a buffer layer (typically Silica or Spin-On-Glass) between the waveguide and the cavity, and multiple lithographic steps to fabricate the waveguide aligned on top of the cavity, which render the fabrication relatively more complex. Moreover, this coupling approach could also limit the sensing operation, as the waveguide on top of the 1D PhC partly covers the useful sensing area of the microcavity.


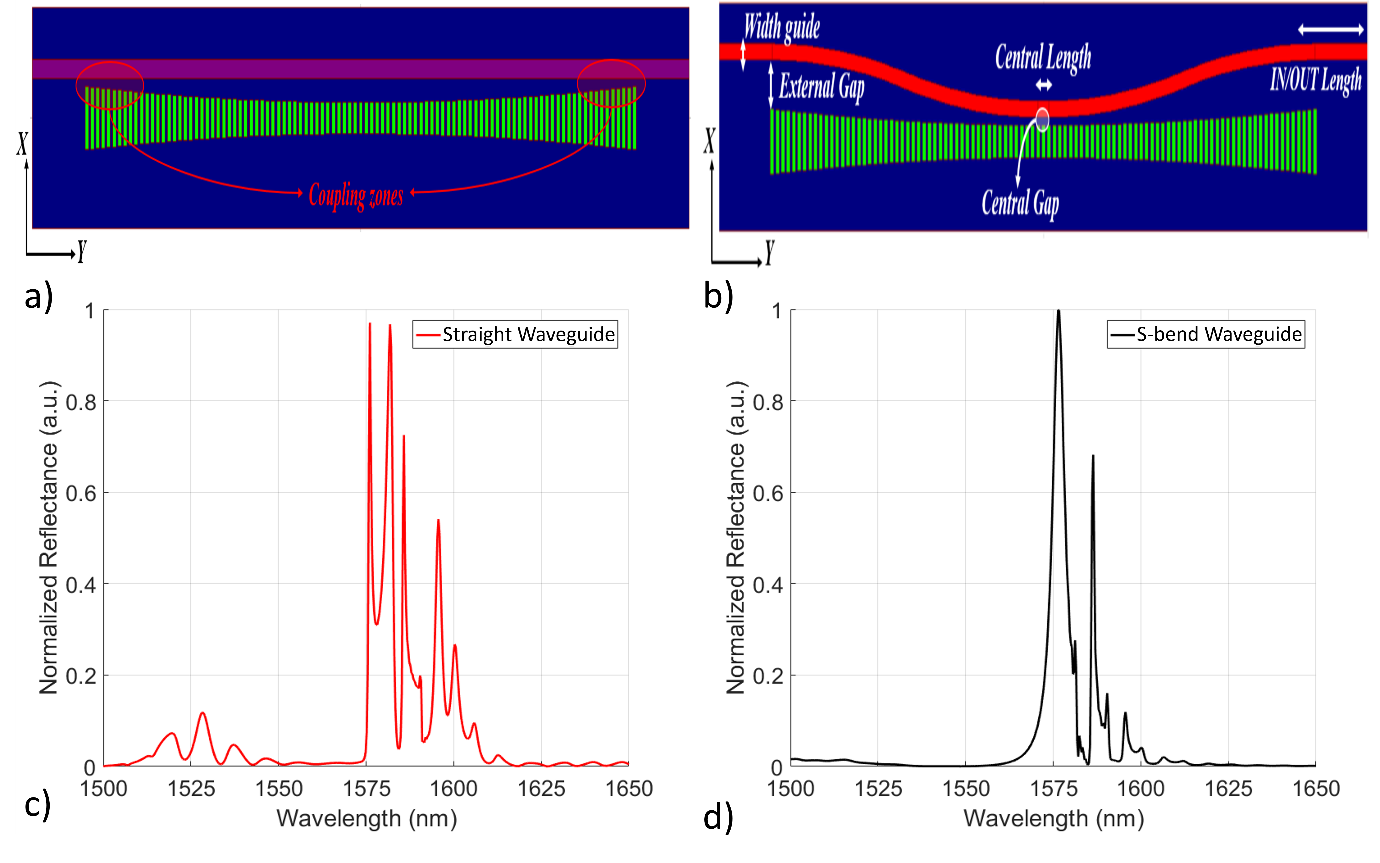


Figure S.6 – a) Schematics of the 1D PhC cavity side-coupled to a straight waveguide and b) schematics of the same 1D PhC cavity side-coupled to an S-bent waveguide limiting the zone of coupling near the centre of the PhC cavity, c) calculated reflection spectrum of the structure in (a) and d) calculated reflection spectrum of the structure in (b). The spectra show the presence of many unwanted modes for the structure in (a) due to the coupling of light on the edges of the cavity, which are not present for structure (b).

The simplest side-coupling approach, shown in **Fig. S.6a**, consists of a straight waveguide laterally separated from the PhC cavity by a gap that sets the coupling strength and the Q-factor of the waveguide-cavity integrated system (loading Q-factor). However, this geometry favours the evanescent coupling of the guided mode of the waveguide into multiple modes at the edges of the PhC cavity, increasing spectral noise. To overcome this problem, the SiN waveguide has been reshaped in a way to enhance the evanescent coupling from the waveguide to the centre of the PhC cavity, as shown **Fig. S.6b**, cleaning the system spectral response from all the unwanted resonances, as depicted in the calculated spectra in the case of a straight waveguide and an S-bend waveguide, in **Fig. S.6c** and **S.6d,** respectively.

# S.3 Waveguide-Microcavity Measurements and Lithographic Tuning

After the fabrication, the stick sidewalls angles have been calculated by measuring the long and short bases ($b_{+}$ and $b_{-}$, respectively) of the sticks cross-sections with a scattering electron microscope (SEM), with the fabricated devices mounted at a $45^{\circ}$ on the sample holder, and using them as values in the following equation:

| $\theta=\arctan\left[ \frac{h}{\frac{\left( b_{+}-b_{-} \right)}{2}} \right]$ | (5) |
| --- | --- |

with $h$ representing the height of the PhC sticks. Finally, in order to evaluate the cavities optical performances in different environments, various upper-cladding configurations have been obtained by spin-coating some of the samples with a 500 nm thick layer of Spin-On-Glass (Accuglass T12), cured at a temperature of $425℃$ and exhibiting n = 1.40 at $\lambda=1550$ nm. Specifically, the fabricated devices were divided into four configurations depending on the upper-cladding refractive index, $n_{upp}$, and stick sidewalls angle $\theta$: 1) $n_{upp}=1.40$ and $\theta=0^{\circ}$, 2) $n_{upp}=1.40$ and $\theta=7^{\circ}$, 3) $n_{upp}=1.00$ and $\theta=0^{\circ}$ and 4) $n_{upp}=1.00$ and $\theta=7^{\circ}$.


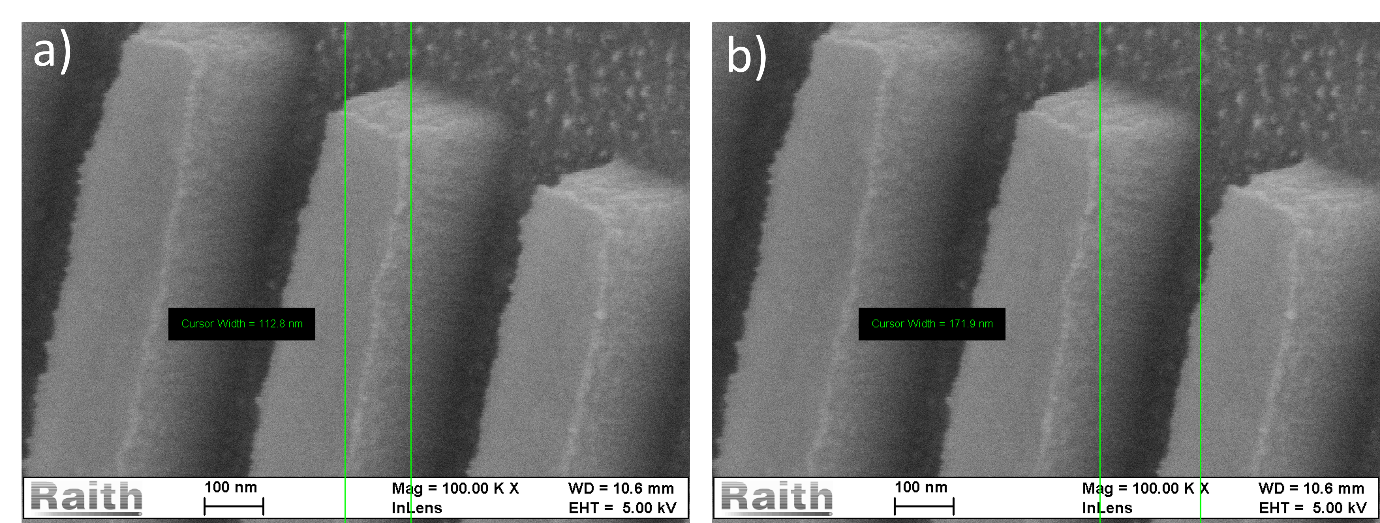


*Figure S.7 – SEM image of one of the 1D PhC cavity etched under 10 mTorr of chamber pressure to aim for a 7° sidewall angle. a) measurement of the top base of the trapezoidal stick and b) measurement of the bottom base of the trapezoidal stick, resulting In a sidewall angle of ~6.7°.*

The transmission time-averaged spectra of all the devices were acquired using the setup in **Fig. S.**8. The fibre-coupled output of an optical broadband source (Amonics ASE, 1500 nm to 1650 nm) is collimated with a NIR optimised 20x lens and then focused onto the sample with a 60x NIR optimised lens mounted onto an XYZ translation stage (used to accurately align the signal to the sample waveguides). The sample is mounted onto an Al holder provided with Al blocks and a nested Peltier element connected to a temperature controller (TEC) to provide chip temperature stability. The output of the sample waveguide is collected via a 60x NIR optimised lens and again collimated towards another 20x NIR optimised lens at the farthest end of the setup, which downsizes the beam for the collection through a fibre. The resulting signal, also containing the spectral information of the microcavity, is then sent to an optical spectrum analyser (OSA) and power meter connected via a fibre-based beam splitter. The utilisation of a polarization beam splitter is also added in the left-side of the optical path, in order to investigate polarisation dependent responses of the waveguide-microcavity systems. To investigate the passive microcavity response with an analyte solution (effectively changing the upper-cladding RI seen by the microcavity), a dropper is used to deposit droplets of solutions with a known concentration (required to extrapolate the experimental sensitivity of the device). After every drop and measure, the sample is unmounted, thoroughly cleaned with IPA and Acetone and dried carefully with an air gun.


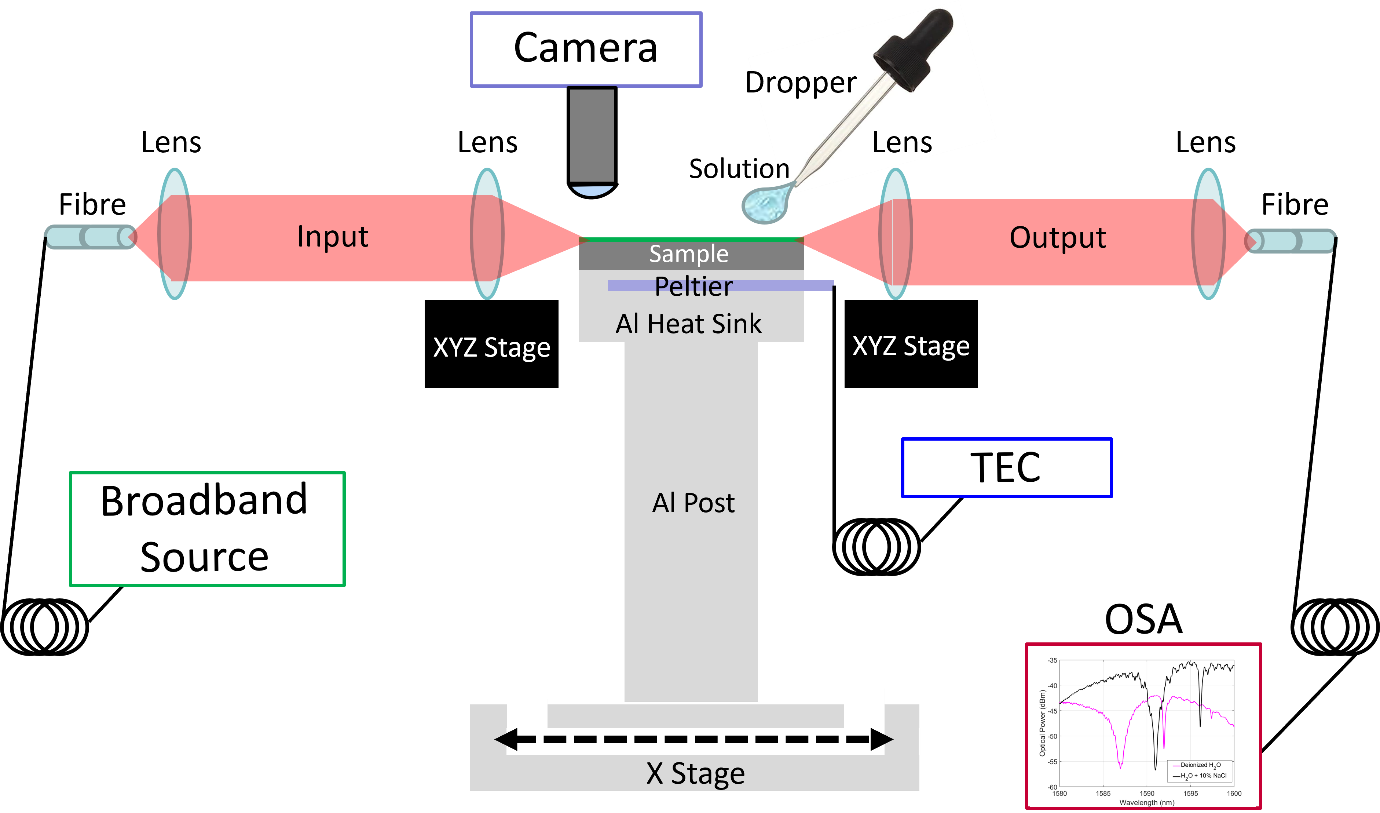


Figure S.8 – Experimental setup used to characterise the passive waveguide-microcavity systems on the SiN chips.

The quadratically tapered devices have been fabricated in sets, in which different cavity geometry parameters are swept to achieve lithographic tuning. The parameters varied in each sets include the stick width $W_{x}$ (228 nm, 248 nm, 268 nm, 288 nm and 308 nm), the side-coupling gap (100 nm, 250 nm and 450 nm) and waveguide coupling shape (straight, curved with a radius of 100 µm, curved with a radius of 50 µm), while always maintaining the cavity stick heights fixed at $W_{y}(N)$ = 2.0 µm and $W_{y}(0)$ = 1.4 µm


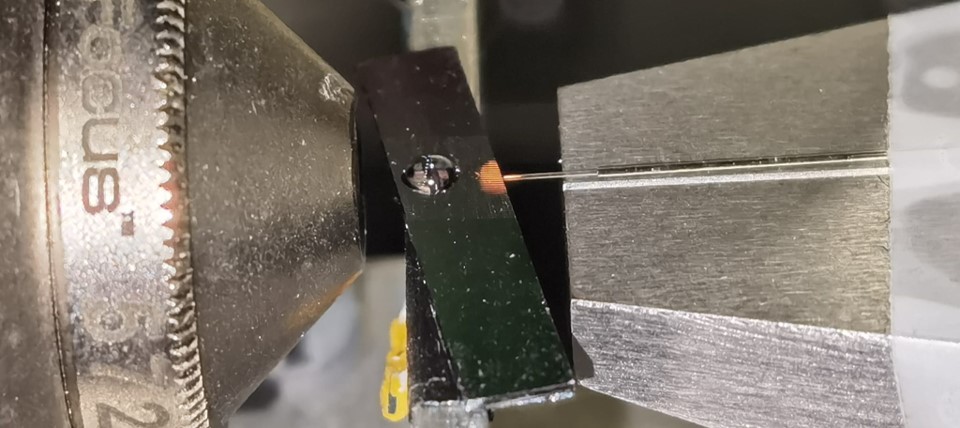


Figure S.9 – Photo of the chip with waveguide-cavity devices mounted on the setup for the sensing measurement.

**Fig. S.9** shows a photo of the setup, in which a chip is mounted on the Al sample-holder for the sensing measurements. A lens is present for the input, while a lensed-fibre is used for the output in this case (interchangeable with the system of lenses shown on the left-side of **Fig. S.8**). A droplet of solution is sitting on the chip, completely covering the microcavities under measurement. All the waveguides belonging to the lithographic tuning sets are also visible in the picture.

The spectral response of one of the devices is shown in **Fig. S.10a**, with optical resonances manifesting as peaks on the magenta curve representing the normalized reflection (R) of the cavity and dips in the blue curve representing normalized transmission (T). **Fig. S.10b** shows the measured spectra of the same cavity design in the case of a straight and a bent waveguide side-coupled to it (as shown in **Fig. S.6a** and **S.6b**), highlighting the suppression of 3 unwanted side modes (circled in black) in the case of the bent waveguide, confirming the predictions of the simulations.


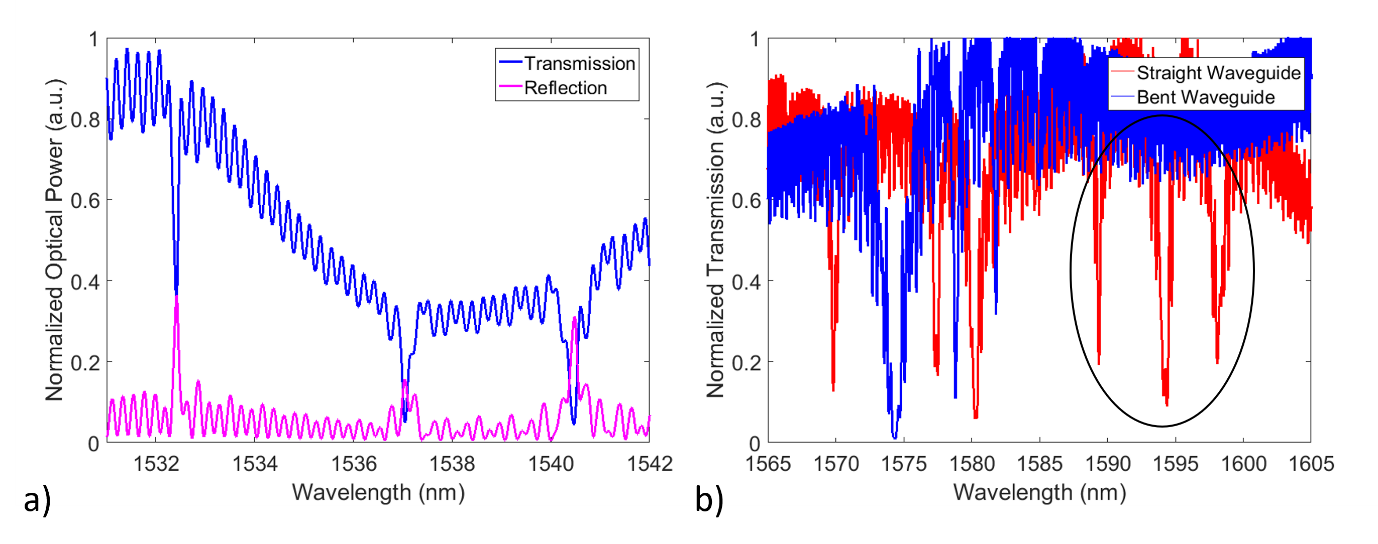


Figure S.10 – a) Normalized Reflection and Transmission spectra of a measured device ($W_{x}$ of 248 nm, side-gap of 250 nm and $a$ of 480 nm, curved waveguide) and b) Normalized transmission spectra of measured devices with the same cavity design but different side-coupling conditions: the red curve corresponds to side coupling with a straight waveguide while the blue curve corresponds to the side-coupling with an S-bent waveguide, clearly showing the elimination of some unwanted resonant modes present in the red curve ($W_{x}$ of 268 nm and $a$ of 476 nm, side-gap of 250 nm).

Lithographic tuning of the cavity has also been carried out, in order to evaluate the influence of the waveguide-cavity spatial separation (referred to as side-gap) and stick width on the performances of the cavity. The measured spectral response of the same cavity design with different side-gap is shown in **Fig. S.11a**, where a clear spectral broadening of all the cavity resonances is present by reducing the side-gap from 250 nm to 100 nm. This demonstrates the strong dependence of system optical performances on the coupling distance (i.e., the side-gap directly affects the loading Q-factor of the device). **Fig. S.11b** shows the measured spectra of similar cavity designs in which only the stick width is varied, manifesting a wavelength red-shift of the PhC cavity resonances with increasing stick widths, due to the increasing percentage of high index material (SiN) in each period of the structure. The stick width also affects the Q-factor of the device, as deduced from the variation in resonance FWHM in the three cases shown (widths of 228, 248 and 268 nm), and expected from the simulated data.

The cavity ($Q_{0}$) and coupling ($Q_{c}$) Q-factors have been calculated through the measured Q-factor ($Q_{m}$) and transmission extinction ratio ($T$) of each device, according to the equations:

| $Q_{0}=\frac{Q_{m}}{\sqrt{T}}$ | (6) |
| --- | --- |
| $\frac{1}{Qc}=\frac{1}{Q_{m}}-\frac{1}{Q_{0}}$ | (7) |


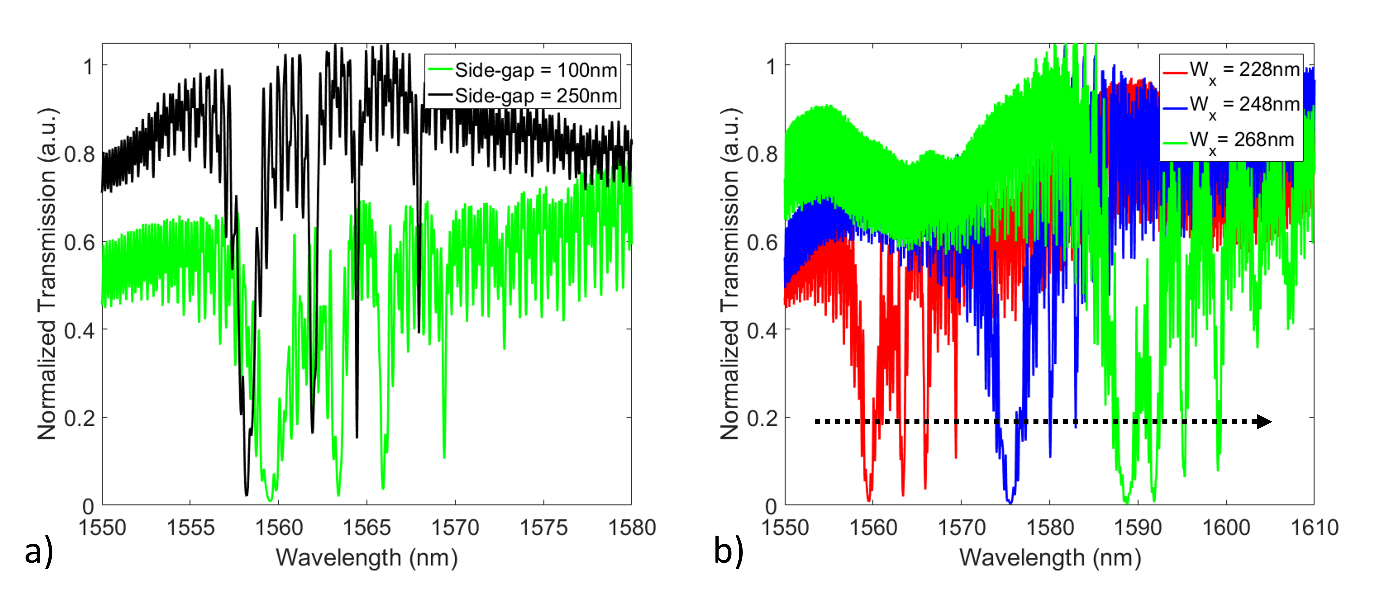


Figure S.11 – a) Measured normalized transmission spectra of different devices with the same cavity design but different side-coupling distance: 250nm (black) and 100nm (green), $W_{x}$ of 248 nm and $a$ of 486 nm and b) Measured normalized transmission spectra of different devices with the same cavity design but different stick widths (Wx): 228nm (red), 248nm (blue) and 268nm (green), side-gap of 100 nm and $a$ of 486 nm. The black dashed arrow represents the resonances wavelength shift with increasing Wx.

# S.4 Solution Concentration to Upper-Cladding RI

The NaCl solution at different % concentrations have also been converted into upper-cladding refractive index values using the technique demonstrated in [60]. The plot of upper-cladding RI against salt concentration is shown in **Fig. S.12**, exhibiting a fairly linear trend (red dashed line).


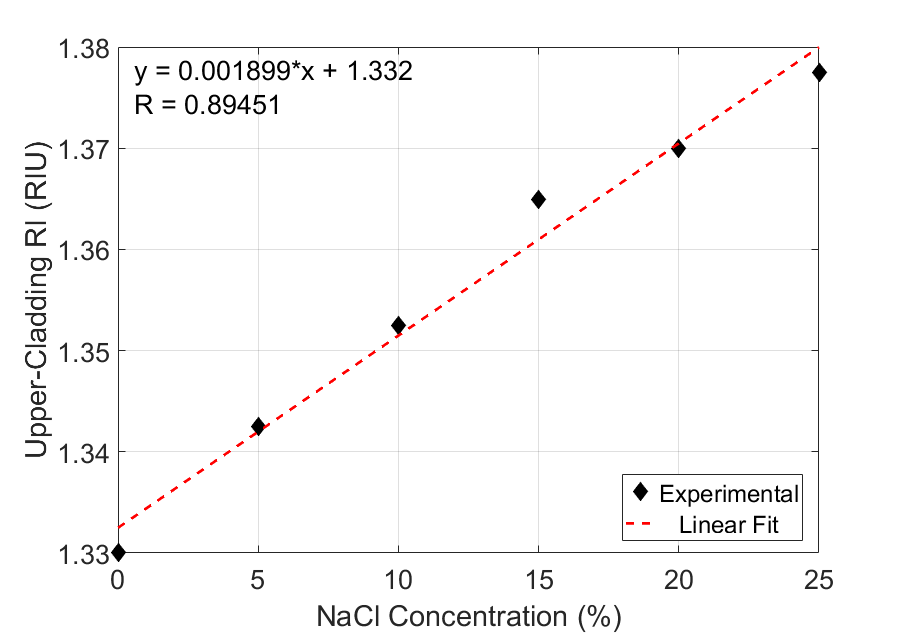


Figure S.12 – Upper-cladding RI against salt concentration in %, in which the black diamonds represent the experimental data while the red-dashed line represents a linear fit

**Fig. S.13** shows the actual resonance wavelength shift with increasing NaCl concentrations, with **Fig. S.13b** zooming onto one of the datapoints and related error bar.


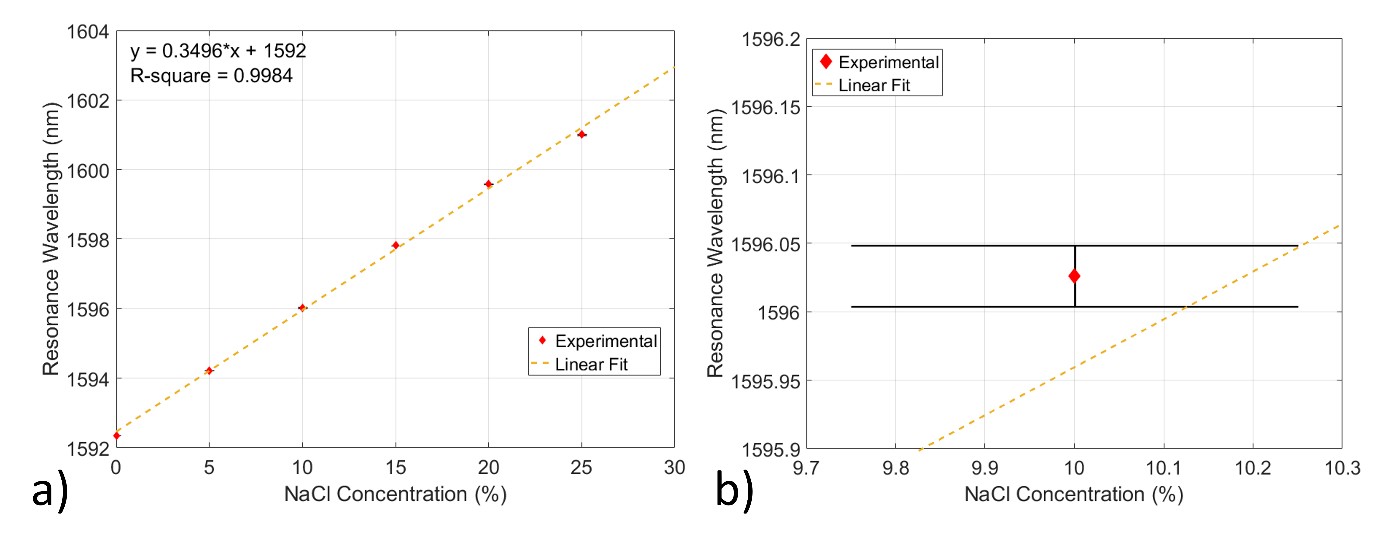


Figure S.13 – a) Shift of the resonance wavelength of the microcavity with salt concentration, plotted with error bars and standard deviations and b) same plot of a) zoomed on one of the datapoints (10% NaCl) to better show the error bar. Yellow dashed lines represent the linear fit of the datapoints.

The measured standard deviation (σ) is 45.3 pm, 56.7 pm, 149.3 pm, 78.7 pm, 71.3 pm, 130.7 pm for the 0%, 5%, 10%, 15%, 20% and 25% NaCl concentrations respectively.

The same sensing measurement has been repeated with a solution of sucrose (C_12_H_22_O_11_), a common form of sugar, dissolved in de-ionised water (DIW) at different concentrations. The repeated measurements of the microcavity spectrum for DIW and for each solution concentration values are shown in **Fig. S. 14**, with DIW depicted in **Fig. S.14a**


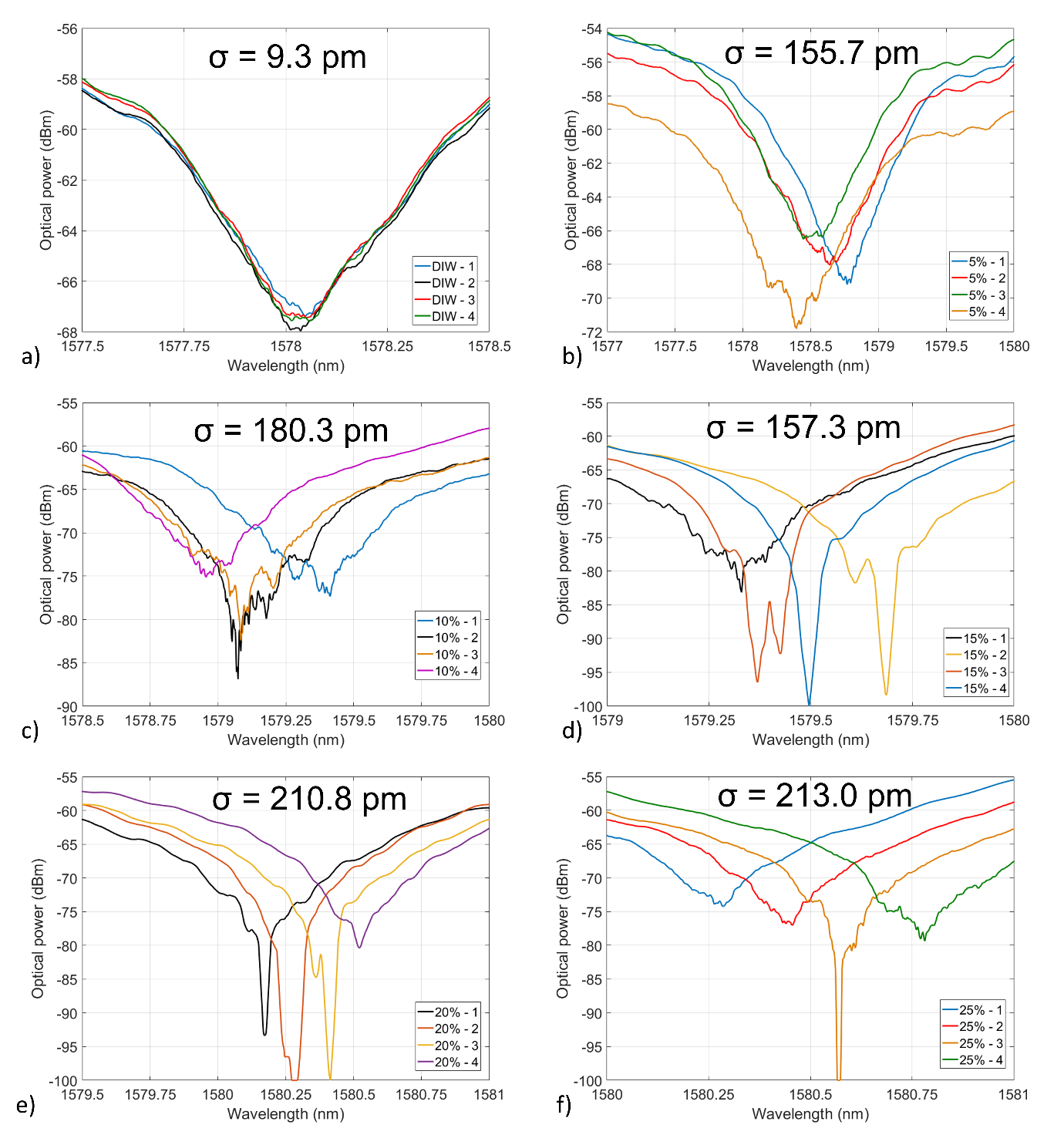


Figure S.14 – Repeated measurements of the microcavity spectrum with different solution: a) de-ionised water, b) 5% sugar, c) 10% sugar, d) 15% sugar, e) 20% sugar and f) 25% sugar. The standard deviation associated to each repeated measurements is reported on each plot.

The standard deviation (σ) associated to the measurements with the sugar solutions is 9.3 pm, 155.7 pm, 180.3 pm, 157.3 pm, 210.8 pm, 213.0 pm for the 0%, 5%, 10%, 15%, 20% and 25% sugar concentrations respectively. Apart for DIW, the standard deviations for the sugar solutions are slightly higher than the ones for NaCl.


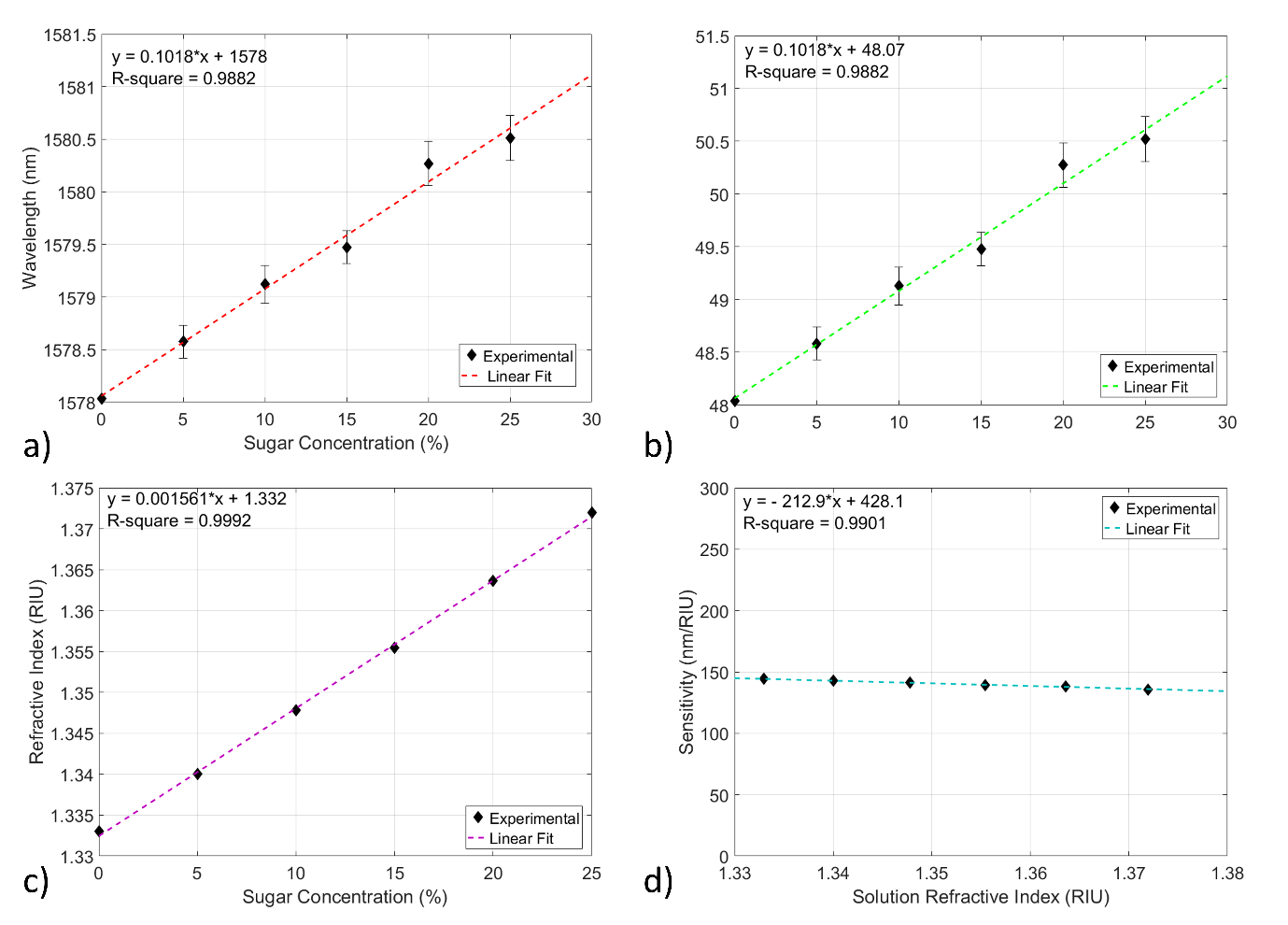


Figure S.15 – a) Plot of the measured microcavity resonance wavelength against sugar concentration with error bars. The red-dashed line represents a linear fit of the experimental data, b) plot of the resonance wavelength shift ($\Delta\lambda$) with increasing sugar concentration, green-dashed line represents a linear fit, c) plot of the refractive index of the solution with sugar concentration, with magenta-dashed line representing a linear fit and d) plot of the measured sensitivity with solution refractive index, with turquoise-dashed line representing the linear fit of the datapoints

The linear microcavity resonance wavelength with increasing sugar concentration is plotted in **Fig. S.15a**, with **Fig. S.15b** depicting the measured resonance wavelength shift with the sugar concentration in respect of the air-cladded cavity resonance. **Fig. S.15c** shows a plot of the solution refractive index with the sugar concentration (as according to [60]) and **Fig. S.15d** depicts the passive microcavity sensitivity (S) with increasing sugar (sucrose) concentration, measured to be ~150 nm/RIU in the sugar concentration range investigated. The detection limits for the passive microcavities used with NaCl and C_12_H_22_O_11_ solutions can also be evaluated using [61], using the measured Q-factors and Sensitivities. The detection limit can be defined as:

| $DL=\frac{R}{S}=\frac{\left( \frac{\lambda}{Q_{meas}} \right)}{S}$ | (8) |
| --- | --- |

With R representing the sensor spectral resolution (i.e., minimum resonance shift measurable, depending of its linewidth) and $\lambda$ the resonance wavelength of the microcavity cladded by a solution with 0% concentration of analyte (DIW in this case). With a Q-factor of 5048 and S of 180 nm/RIU (**Fig. 8d**) for the microcavity used for sensing NaCl, a DL of $1.7\cdot{10}^{-3}$ RIU. For the microcavity used as sensor with the sugar solution, with a Q-factor of 9074 and S of 150 nm/RIU, the detection limit is instead $1.1\cdot{10}^{-3}$ RIU. Which accounts for the broadening of the linewidths of the optical resonances. due to fabrication disorder, small laboratory temperature variations and solution droplets slowly evaporating on the sample. The optimisation of all these variables (lower losses due to fabrication, packaged system with microfluidic channels) could potentially lower the detection limit down to values closer to the calculated ones. Another way to lower the experimental detection limit is to greatly increase the Q-factors of the devices by achieving lasing, as discussed with HECLs in section **S.5**.

# S.5 Hybrid External Cavity Laser

With the refractive index sensor concept described in **section 3** in mind, the fabricated cavities have been butt-coupled to an AlInGaAs rSOA in HECL configuration, and the laser characteristics have been measured and reported in **Fig. S.16**.


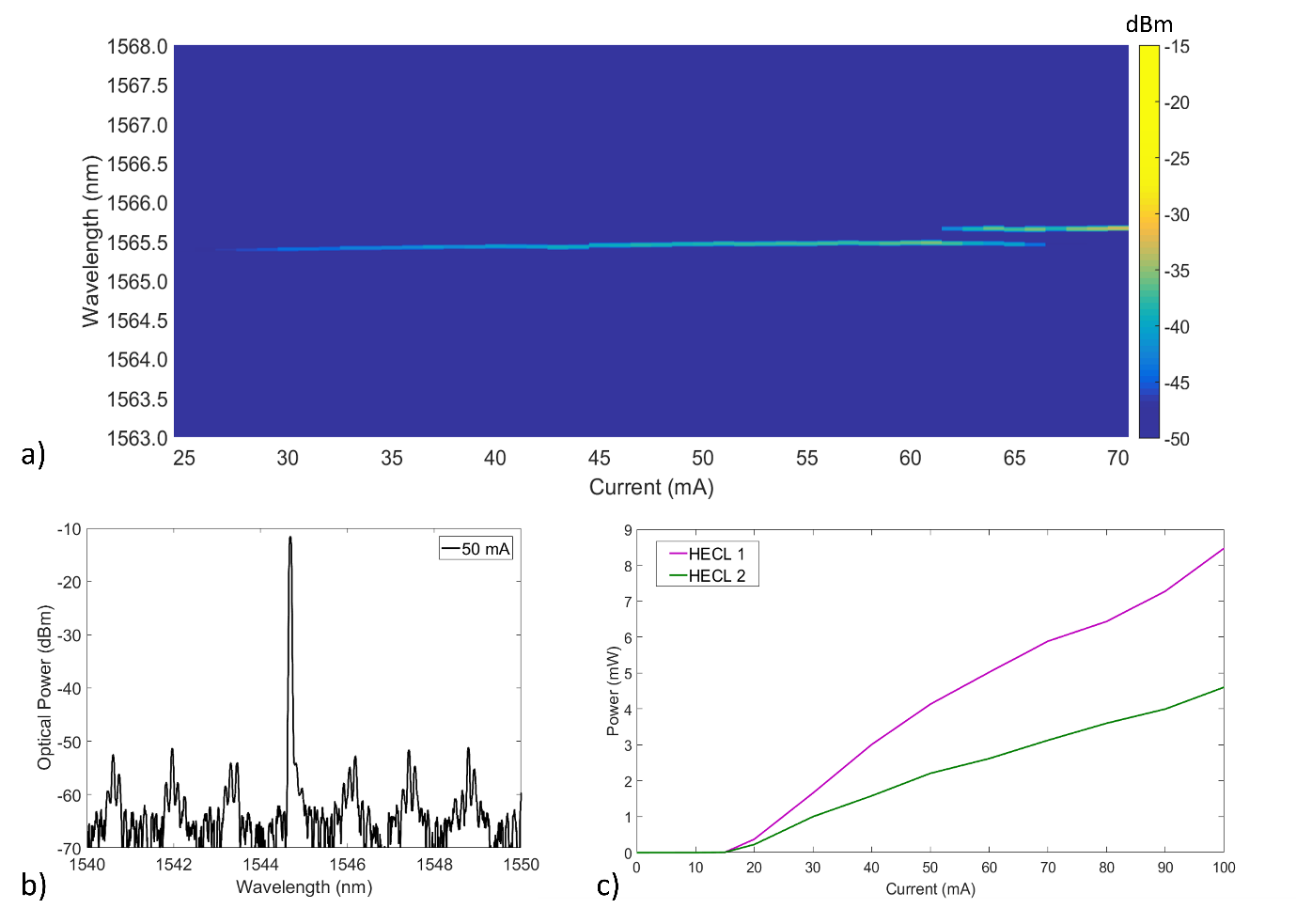


Figure S.16 – a) False colour plot of the optical spectrum of the laser, averaged in time, with the increasing driving current, b) transmission spectrum of the laser taken with a driving current of 50mA and c) laser power against current (L-I) curves of two different HECLs based on the designed 1D PhC cavities.

As shown in **Fig. S.16a**, mode-hop free single-mode regime has been obtained for a large range of driving currents, from 27 to 65 mA. The SiN 1D PhC cavity based HECLs also achieved a relatively low lasing threshold in the range of 27 mA, with side-mode suppression ratios (SMSR) in the range of 40 dB along all the single-mode regimes, as shown in **Fig. S.16b**, where laser spectra of one of the 1D SiN PhC cavity-based HECLs is plotted, for a current of 50 mA. A relatively high-power output in the range of a few mWs have been achieved in this type of HECLs, as depicted for two different laser devices in **Fig. S.16c**. Furthermore, the lasing spectra of the air-cladded devices have been measured with and without the presence of a droplet of de-ionised water on top of the 1D PhC cavities, with the sample maintained at $T_{room}=20.1℃$, depicted in **Fig. 10**, showing a clear lasing wavelength shift in the different upper cladding conditions (corresponding to the microcavity resonance shifts, superimposed in the graph), following the concept depicted in the schematics of **Fig. S.17**.

**
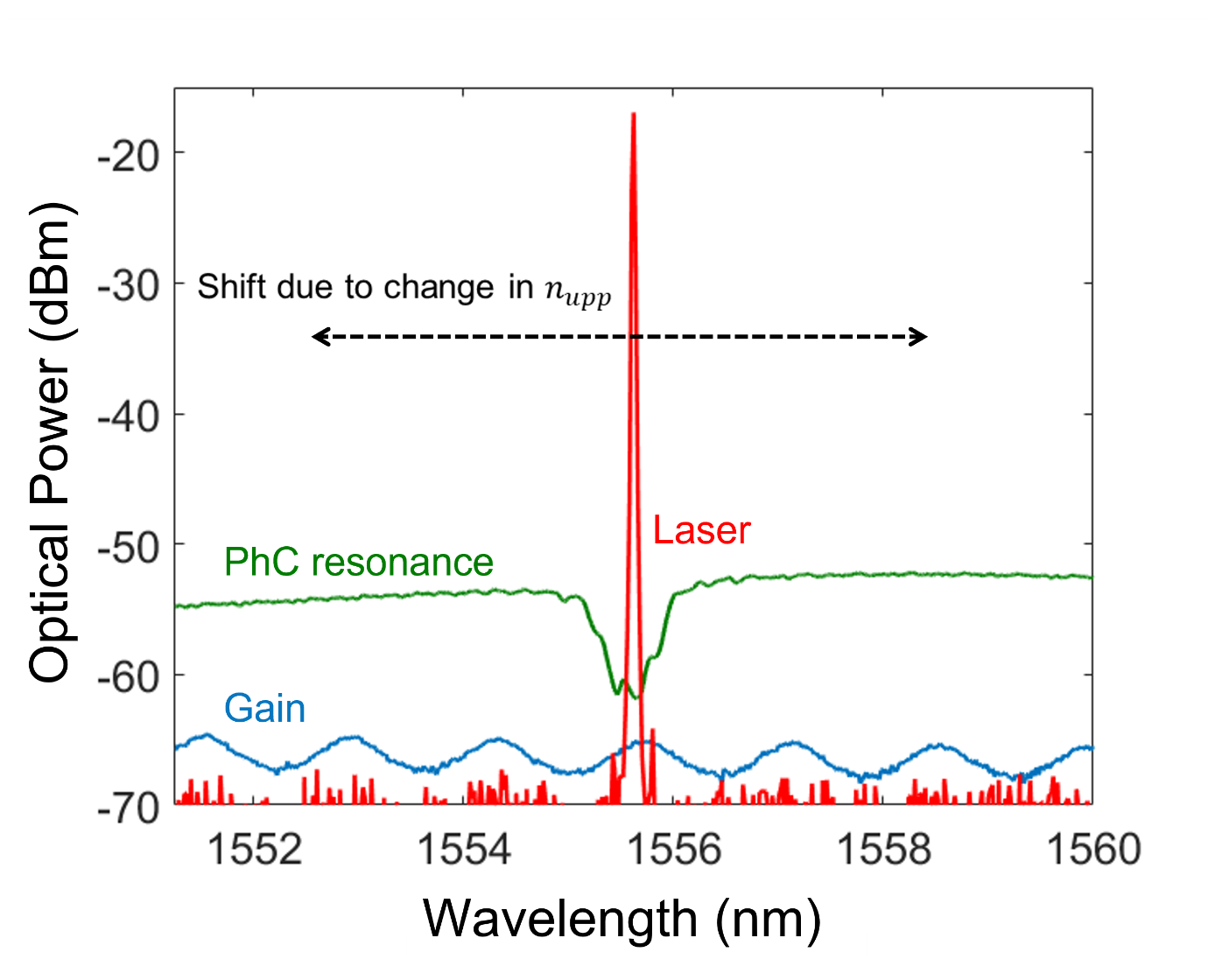
**

Figure S.17 – Schematics of operation of such HECL lasing operation with different upper-cladding solutions, in which the laser wavelength is obtained by the overlap between the laser cavity longitudinal modes with the gain ripple and PhC resonance. The lasing wavelength shifts with the shifting of the PhC cavity resonance, as in the case of the PhC submerged in an analyte solution.

The HECL device is then shown to operate as RI sensor in the schematics of **Fig. S.18**. Both the RSOA and the 1D PhC cavity chips are aligned in butt-coupling configuration through the use of XYZ translational stages until lasing occurs. The translational stage of the RSOA is also provided with pitch and yaw angle adjustments components, in order to maximize the coupling between the chips. Both translational stages are equipped with custom temperature controllers composed by Peltier elements and Al blocks. The temperature of both chips is maintained at 20°C and the laser output is collected by a lensed fibre aligned with facet of the SiN waveguide and the signal is read by an optical spectrum analyser (OSA). A drop of solution is manually deposited on the passive chip via a dropper or pipette (making sure no liquid enters in contact with the RSOA, damaging it). Once the results for a particular solution are collected, the passive chip is washed with IPA and dried thoroughly with an air gun, before repeating the measurement with another drop of solution with different concentration.


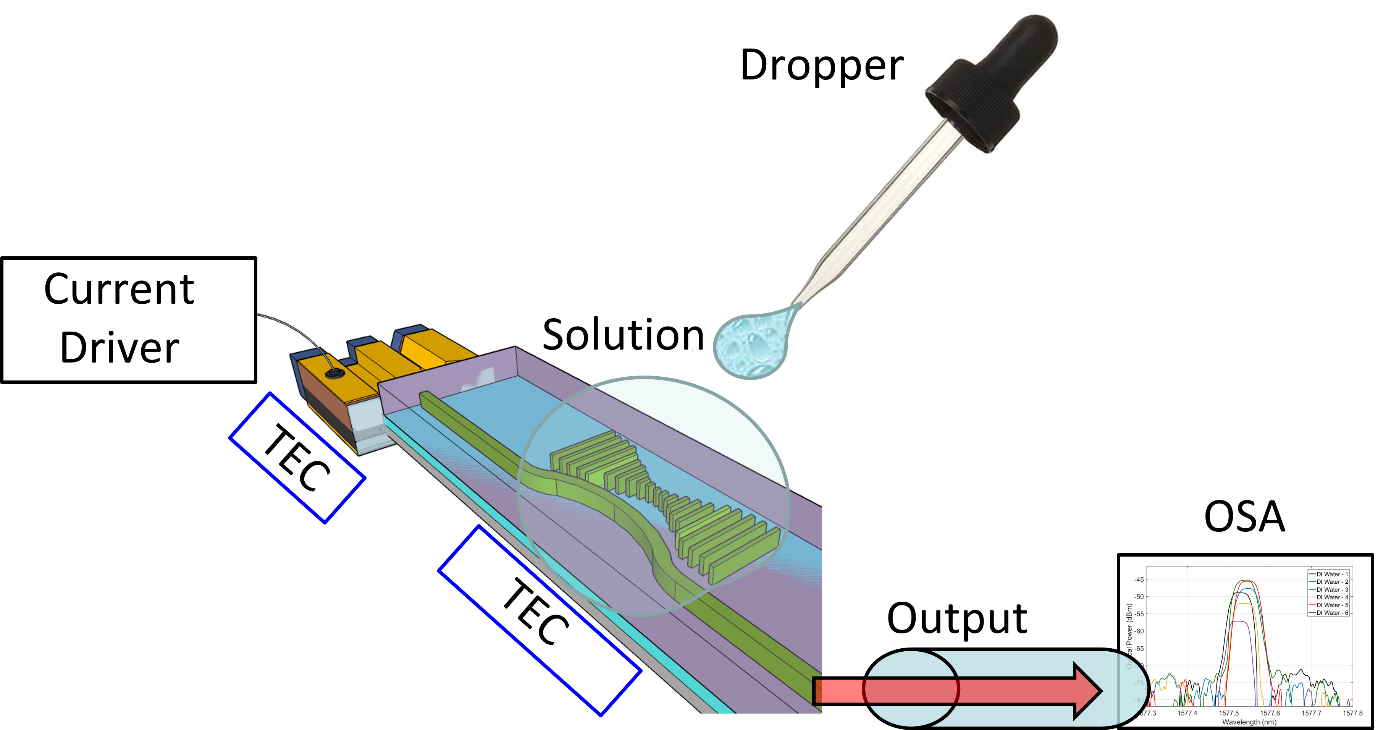


Figure S.18 – Schematics of the HECL based RI sensor measurement, on which the different solutions are drop-cast with a manual dropper.

The complete spectra of the HECL with the different upper-cladding conditions (Air, DI Water, Sugar Solution with increasing concentrations) are shown in **Fig. S.19**, in which the lasing wavelength shifts with the upper-cladding refractive index from air to de-ionised water, to the increasingly concentrated sugar solutions.


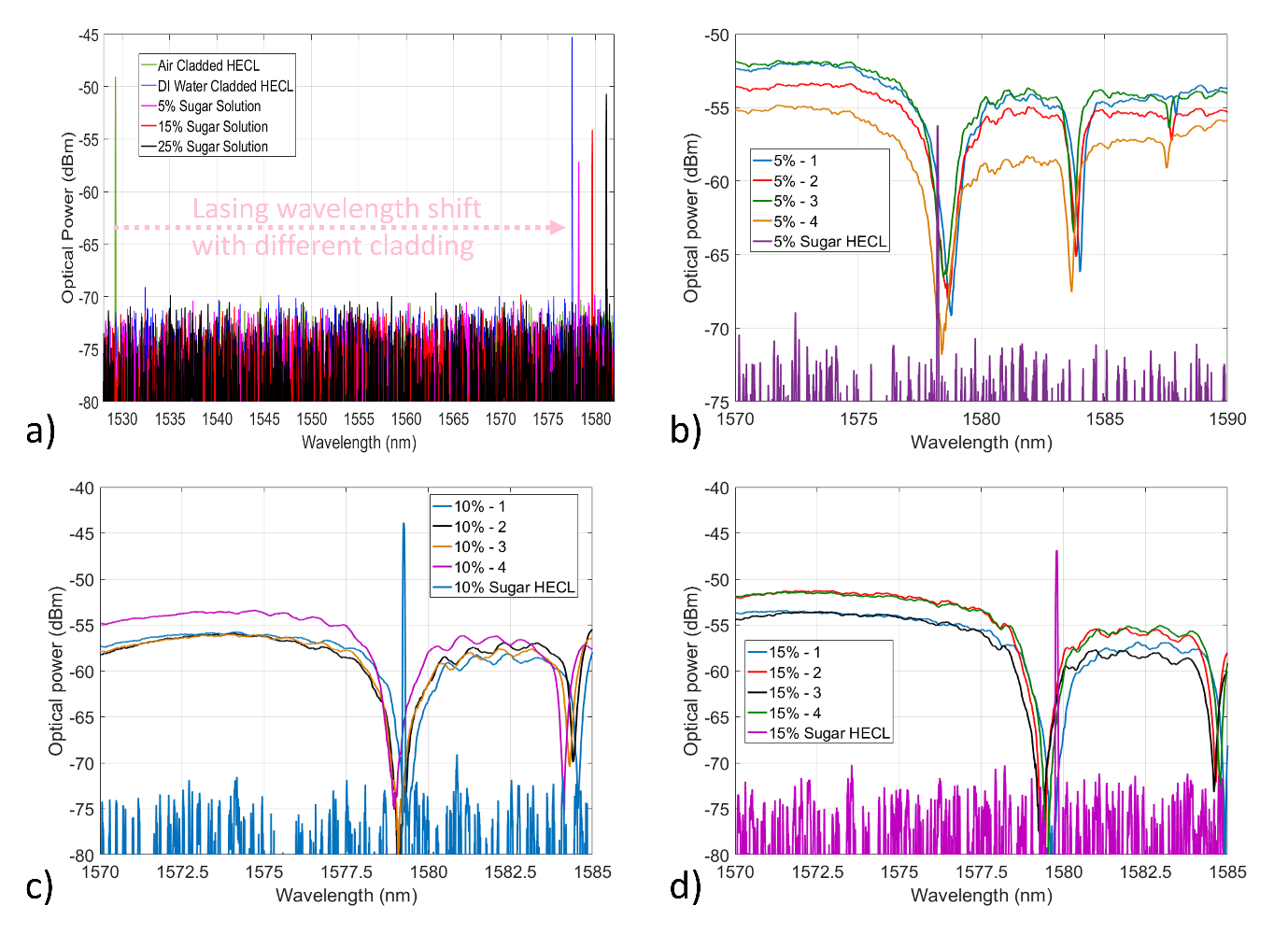


Figure S.19 – a) Lasing spectra of the HECL with different upper-cladding configurations: air (green), DI water (blue), 5% sugar (magenta), 15% sugar (red) and 25% sugar (black), b), c) and d) correspond to the measured HECL lasing spectra superimposed to the measured passive microcavity spectra for solutions with 5%, 10% and 15% sugar respectively.

Multiple measurements of the HECL spectra have been taken, in order to verify the laser stability and sensitivity with the different solutions and the results are shown in **Fig. S.20**. In **Fig. S.20a**, the different measurements of the HECL lasing peaks in the presence of a DI water droplet on top of the 1D PhC cavity are depicted, with **Fig. S.20b** plotting the lasing wavelength shift between measurements, resulting in a mean lasing wavelength of 1577.5370 nm (from a mean lasing wavelength of 1529.2740 nm in the air-cladded case) and a standard deviation of 0.0088 nm.


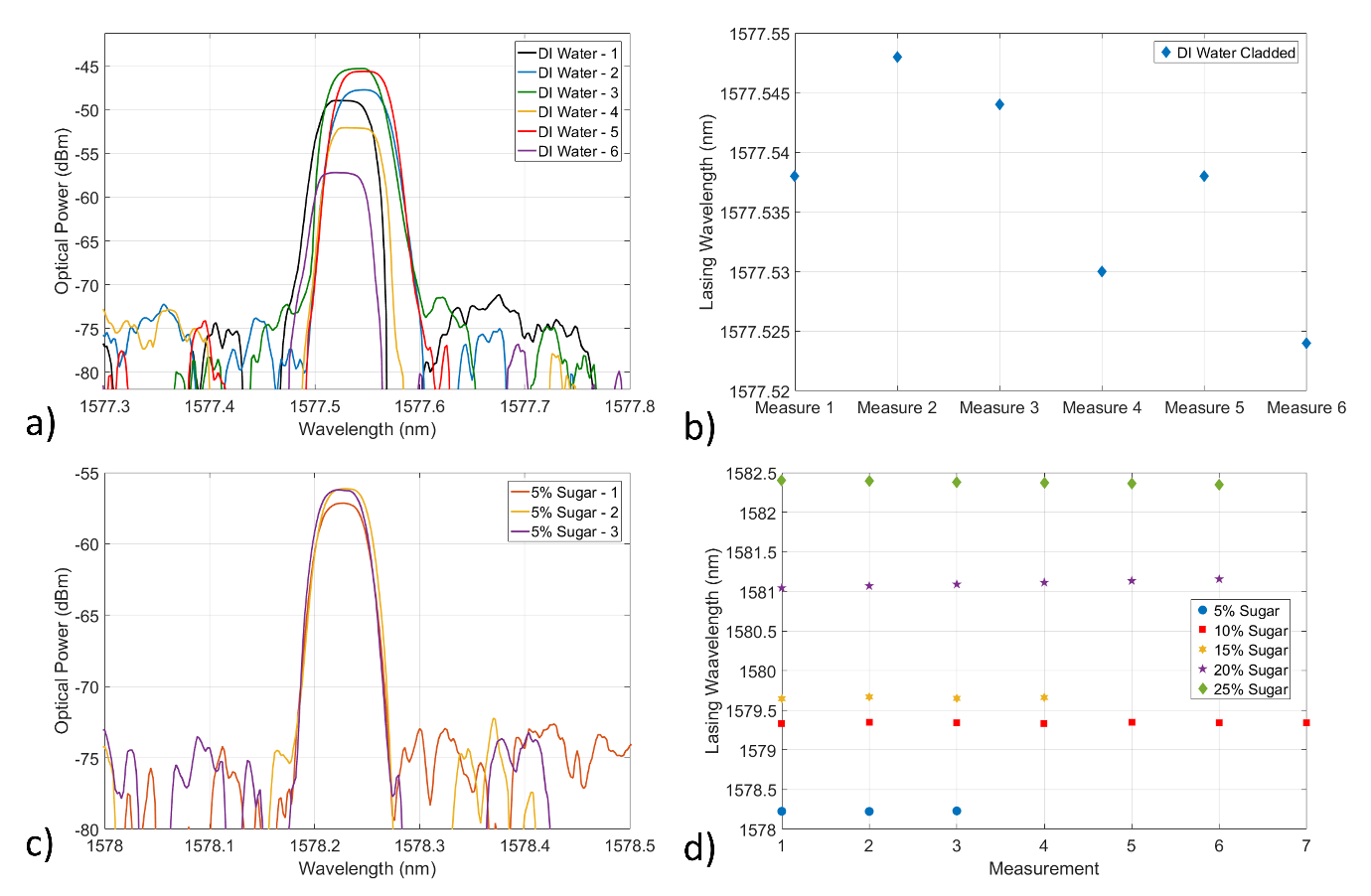


Figure S.20 – a) multiple measurements of the lasing spectrum of the HECL in the presence of DI Water, b) Plot of the lasing wavelength dispersion between the measurements of (a), leading to a standard deviation of 8.8 pm, c) multiple measurements of the lasing spectrum of the HECL in the presence of a 5% concentrated sugar solution and d) lasing wavelength dispersions of the same HECL in the presence of sugar solutions at different concentrations (0%, 5%, 10%, 15%, 20% and 25%), leading to standard deviations of 8.8 pm, 3.1 pm, 6.7 pm, 10.2 pm, 41.4 pm and 20.6 pm respectively.

The same HECL device, then, shifts its lasing wavelength to the mean value of 1578.2267 nm with a standard deviation, σ, of 0.0031 nm with the 5% sugar solution (**Fig. S.20c**), to the mean lasing wavelength of 1579.3391 nm (σ = 0.0067 nm) for the 10% sugar solution, 1579.6565 nm (σ = 0.0102 nm) for the 15% sugar solution, 1581.1010 nm (σ = 0.0414 nm) for the 20% sugar solution and 1582.3773 nm (σ = 0.0206) nm for the 25% sugar solution. The consecutive laser measurements of the same HECL with the different sugar solutions are shown in **Fig. S.21.**


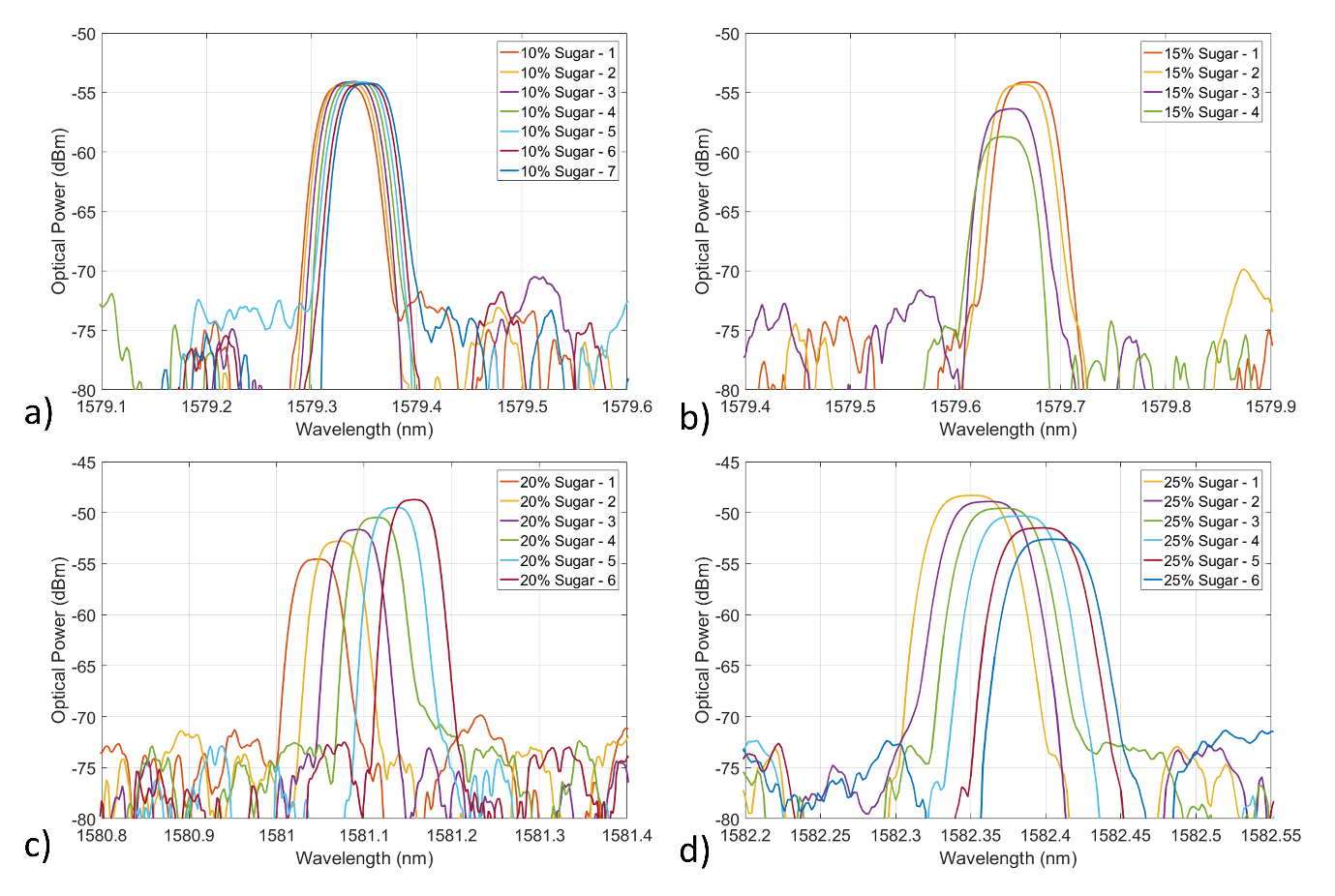


Figure S.21 – Repeated laser measurements of the same HECL in the presence of differently concentrated sugar solution, specifically a) 10% concentration, b) 15% concentration, c) 20% concentration and d) 25% concentration.

The solution-cladded HECL wavelength shift from the air-cladded HECL lasing line with increasing sugar concentrations is plotted in **Fig. S.22a**, while **Fig S.22b** shows the experimental sensitivity associated to the HECL sensor, similar to the associated passive microcavity, in the range of 150 nm/RIU.


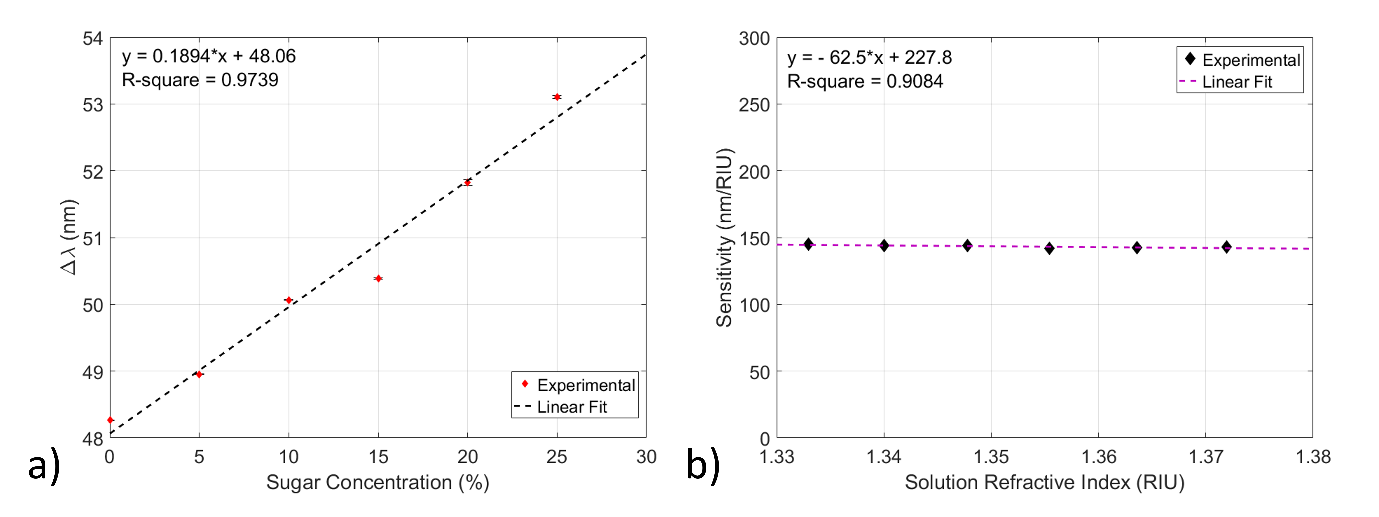


Figure S.22 – a) error bar plot of the lasing wavelength shift from to the air-cladded HECL line with increasing sugar concentrations, the black-dashed line corresponds to a linear fit and b) plot of the experimental sensitivity against solution RI, magenta-dashed line corresponds to a linear fit.

However, due to the HECL lasing line (associated to a Q-factor > 1.1·10^5^, limited by the OSA resolution) being much narrower than the passive microcavity resonance linewidth, the HECL sensor is characterised by an experimental detection limit ~6.6·10^-5^ RIU.

# S.6 1D PhC Cavity Band-Structure

The band structure of the 1D PhC cavity has been numerically investigated, starting from the calculations of the bandgaps corresponding to each ideal 1D PhC composed by an infinite number of repeating sticks with a chosen dimension and shape, parameters that are varied in each different simulation. The simulations have been performed using the FDTD method with the MPB software, simulating one period of the desired PhC in a dipole cloud.

First, the band structure of the same PhC has been calculated with different simulation mesh size to verify the simulation settings for a correctly resolved result. The simulations have been performed with 10, 20 and 40 mesh cells (px) per lattice period (a) and the results are shown in **Fig. S.23a**, in which the 20 px/a appears to be the best setting in terms of resolution and calculation power and speed. The complete band-structure of each PhC has been calculated TE and TM modes in the reduced Brillouin zone, as shown in Fig. S10b. For more clarity of the following plots, only the TE modes are shown from now on.


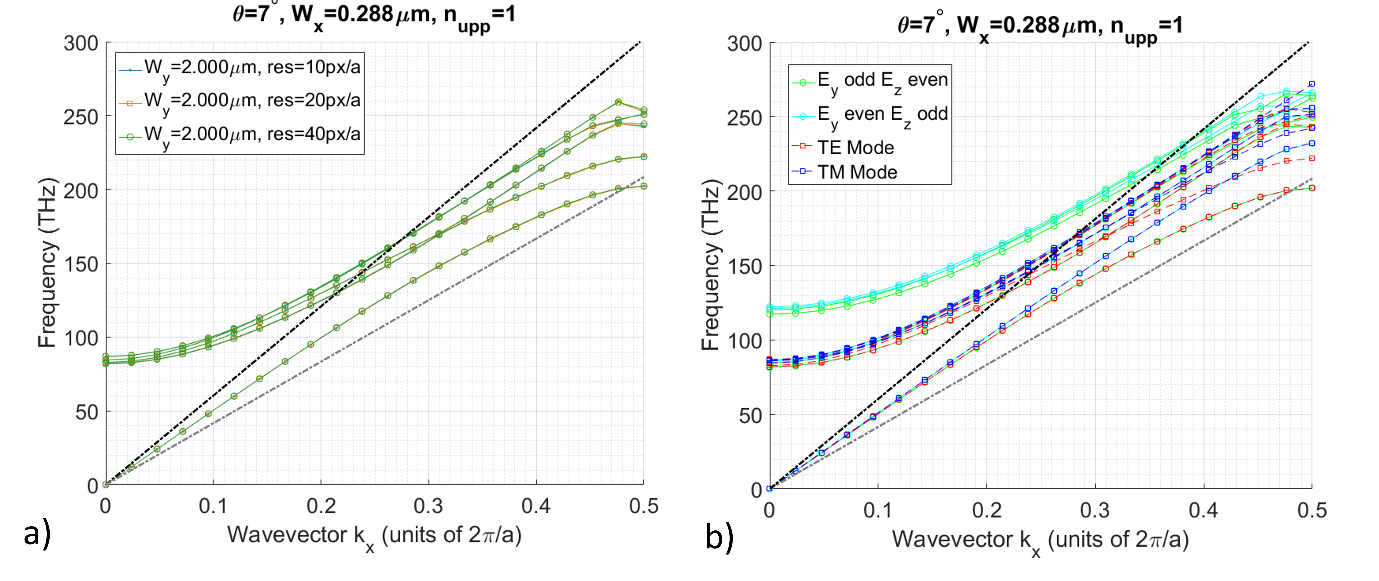


Figure S.23 – a) Band-structure of the 300 nm thick SiN PhC with a = 496 nm, W_y_ = 2000 nm and W_x_ = 288 nm (n_upp_=1.00, $\theta=$7°), with different simulation mesh sizes (10, 20 and 40 px/a) and b) Band-structure for both TE and TM modes of the same PhC in a). The black dashed line corresponds to the light line. Black and grey dashed lines correspond to the air and SiO_2_ light-line respectively

The band structure of the PhC has been calculated, for each sidewall angle ($\theta^{\circ}$, $5^{\circ}$ and $7^{\circ}$), with varying stick width $W_{x}$, to evaluate the width of the bandgap and its shifting in frequency with the increasing stick widths. The calculations of these bandgaps with varying $W_{x}$ are shown in **Fig. S.24**, for upper-cladding refractive indexes of 1 and 1.45. As depicted, the bandgaps shift towards lower frequencies with increasing stick widths ($W_{x}$) in both upper-cladding RI cases, as the effective refractive index of the structure increases (increasing high index material in the period for each increasing value of $W_{x}$).


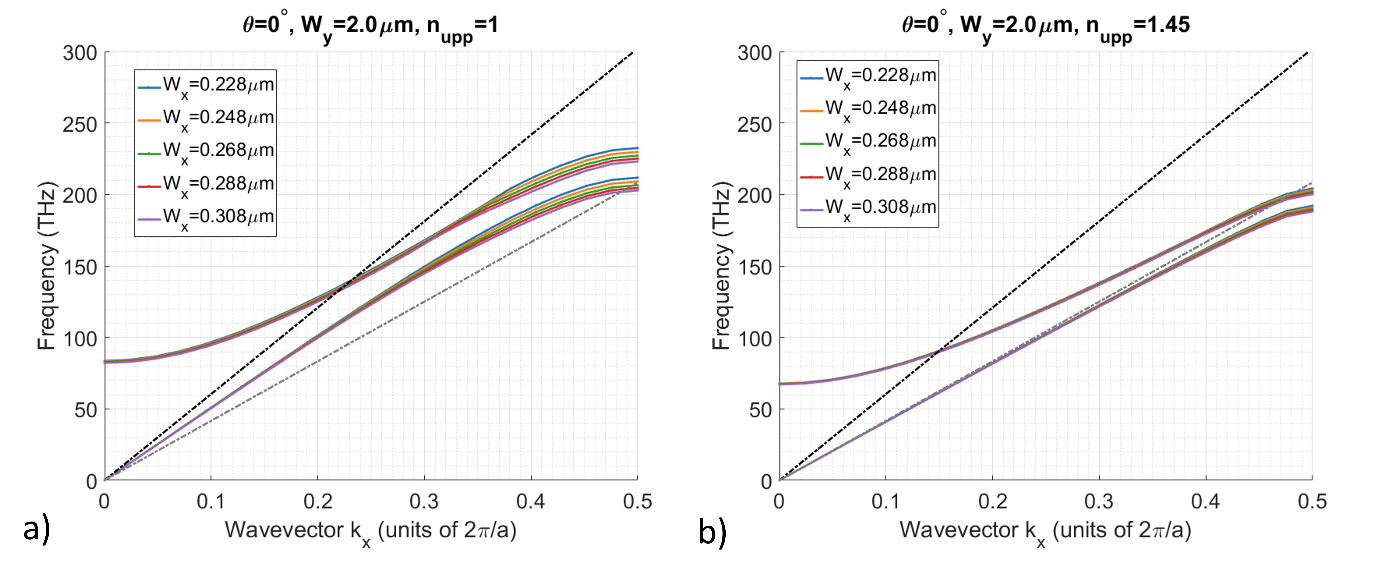


Figure S.24 – a) Band-structures corresponding to 300 nm thick SiN PhCs with a = 496 nm, W_y_ = 2000 nm and increasing W_x_ (n_upp_=1.00, $\theta=$0°) and b) Band-structures corresponding to 300 nm thick SiN PhCs with a = 496 nm, W_y_ = 2000 nm and increasing W_x_ (n_upp_=1.45, $\theta=$0°). Black and grey dashed lines correspond to the air and SiO_2_ light-line respectively

Then, the changes in the 1D PhC bandgap have been investigated with the varying stick height, $W_{y}$, to evaluate the effect of the stick height $W_{y}$ quadratic tapering along x-axis (from 2000 nm at the edges to 1400 nm at the centre of the cavity). The numerical results are shown in **Fig. S.25**, for an upper-cladding RI of 1 and 1.45 (**Fig. S.25a**, and **Fig. S.25b** respectively). As shown in the plots, the $W_{y}$ tapering of the stick shifts and narrow the bandgap, which results in the trapping of the modes into the PhC cavity: the propagating modes at the edges (but outside) of the bandgap of the ideal PhC with $W_{y}$ = 1400 nm are falling inside the bandgaps of the PhCs with larger $W_{y}$. This translates into the coupling of supported propagating modes into the centre of the tapered 1D PhC cavity, which are then reflected back and forth inside it, as the rest of the edge sticks (with larger $W_{y}$) act as mirrors – the modes coupled into the cavity centre are forbidden modes for the rest of the PhC (lateral sticks).


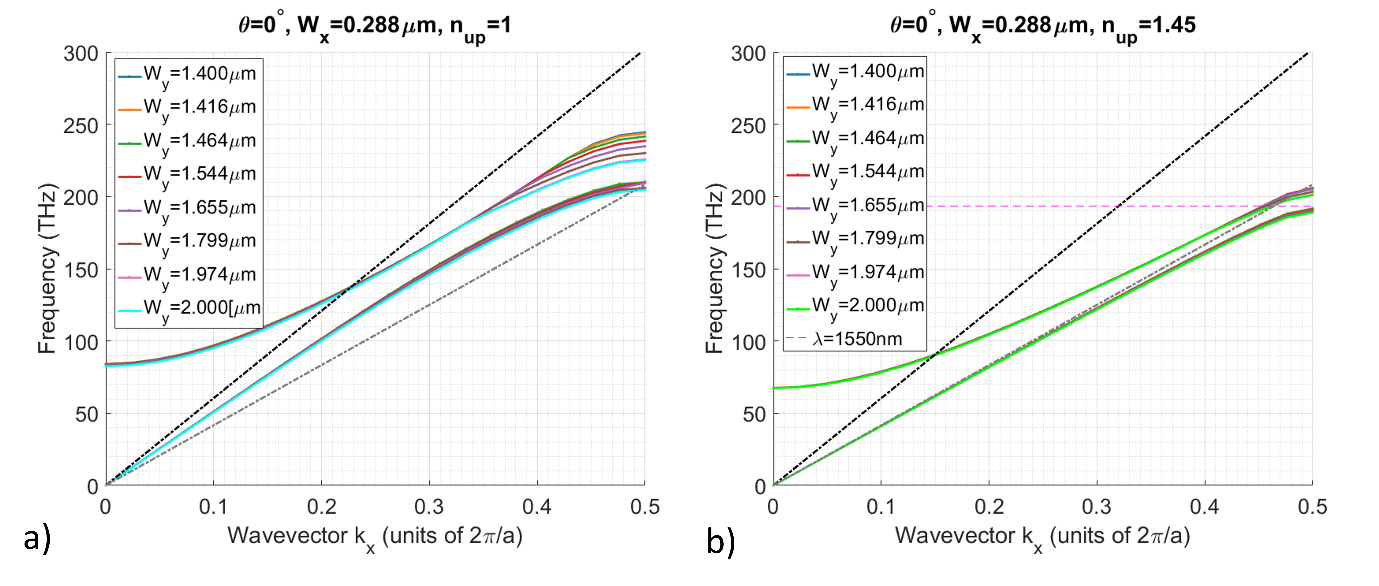


Figure S.25 – a) Band-structure corresponding to 300 nm thick SiN PhCs with a = 496 nm, W_x_ = 288 nm and increasing W_y_ (n_upp_=1.00, $\theta=$0°) and a) Band-structures corresponding to 300 nm thick SiN PhCs with a = 496 nm, W_x_ = 288 nm and increasing W_y_ (n_upp_=1.45, $\theta=$0°). Black and grey dashed lines correspond to the air and SiO_2_ light-line respectively

As expected, the increasing upper-cladding refractive index has a direct effect on the 1D PhC bandgaps, shifting them towards lower frequencies, due to the increase of the structure effective refractive index.


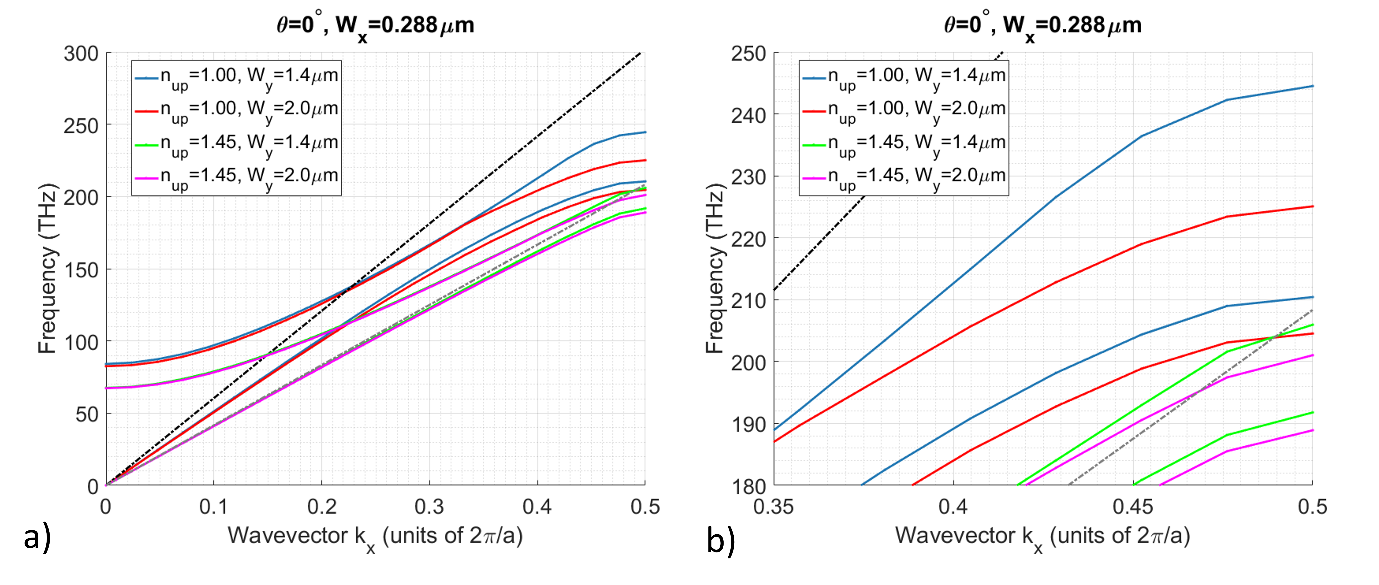


Figure S.26 – a) Band-structure corresponding to the 300 nm thick SiN PhCs with a = 496 nm, W_x_ = 288 nm and W_y_ = 1400 nm and 2000 nm, with different upper-cladding refractive index (n_upp_=1.00 and n_upp_=1.45, $\theta$=0°) and b) zoomed version of the plot in a). Black and grey dashed lines correspond to the air and SiO_2_ light-line respectively

**Fig. S.26** shows the effect of the upper-cladding RI on the structure bandgap. The band-structures for the PhCs with $W_{y}$ = 1400 nm and $W_{y}$ = 2000 nm (corresponding to $W_{y}\left( 0 \right)$ and $W_{y}(i_{max})$ of the tapered 1D PhC cavity) are plotted with different upper-cladding RI (1 and 1.45). The shifting of the bandgap at lower frequencies with increasing upper-cladding RI leads to longer wavelength 1D PhC cavity resonant modes.


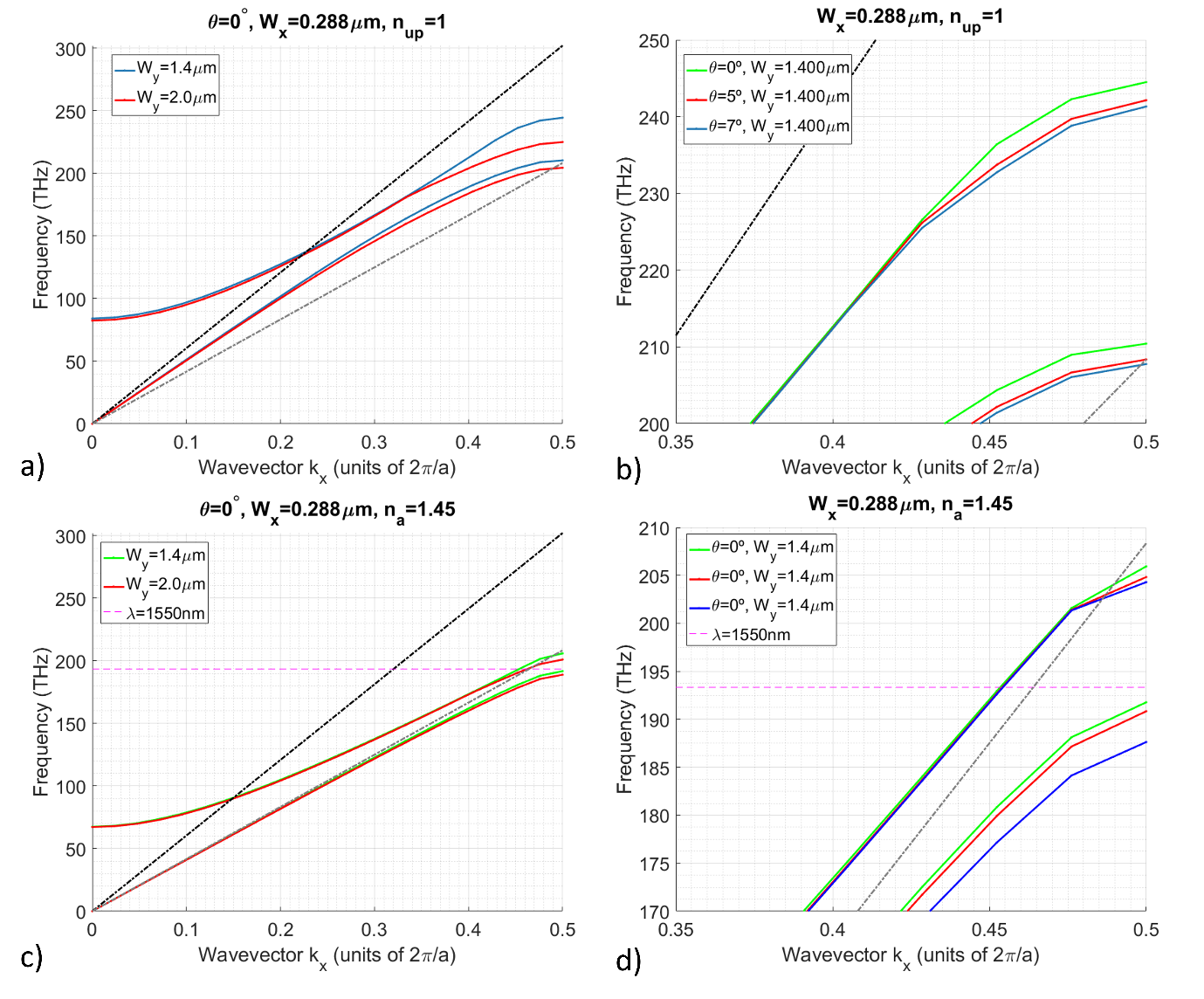


Figure S.22 – a) Band-structure corresponding to the 300 nm thick SiN PhCs with a = 496 nm, W_x_ = 288 nm and W_y_ = 1400 nm and 2000 nm (n_upp_=1.00) and b) zoomed plot of the band-structure of the PhCs similar to (a) (Wy = 1400 nm) with increasing sidewall angle ($\theta$=0°, $\theta$=5°, $\theta$=7°); c) Band-structure corresponding to the 300 nm thick SiN PhCs with a = 496 nm, W_x_ = 288 nm and W_y_ = 1400 nm and 2000 nm (n_upp_=1.45) and b) zoomed plot of the band-structure of the PhCs similar to (c) (Wy = 1400 nm) with increasing sidewall angle ($\theta$=0°, $\theta$=5°, $\theta$=7°). Black and grey dashed lines correspond to the air and SiO_2_ light-line respectively

Finally, the stick sidewall angle effect on the band-structure of the PhCs has been investigated by changing the stick shapes from rectangular cross-sections to the trapezoidal ones ($\theta=0^{\circ}$, $5^{\circ}$ and $7^{\circ}$ respectively). As shown in **Fig. S.22**, the increasing sidewall angle slightly shifts the structure bandgaps to lower frequencies. Moreover, the bandgaps corresponding to trapezoidal sticks are slightly broadened, as more notable in the case of $n_{upp}$ = 1.45 in **Fig. S.22b**. Finally, the calculated bandgaps of the PhCs with increasing $W_{y}$ are shown in the full Brillouin zone in **Fig. S.23**, related to the zoomed version in **Fig. 3b** of the main text.


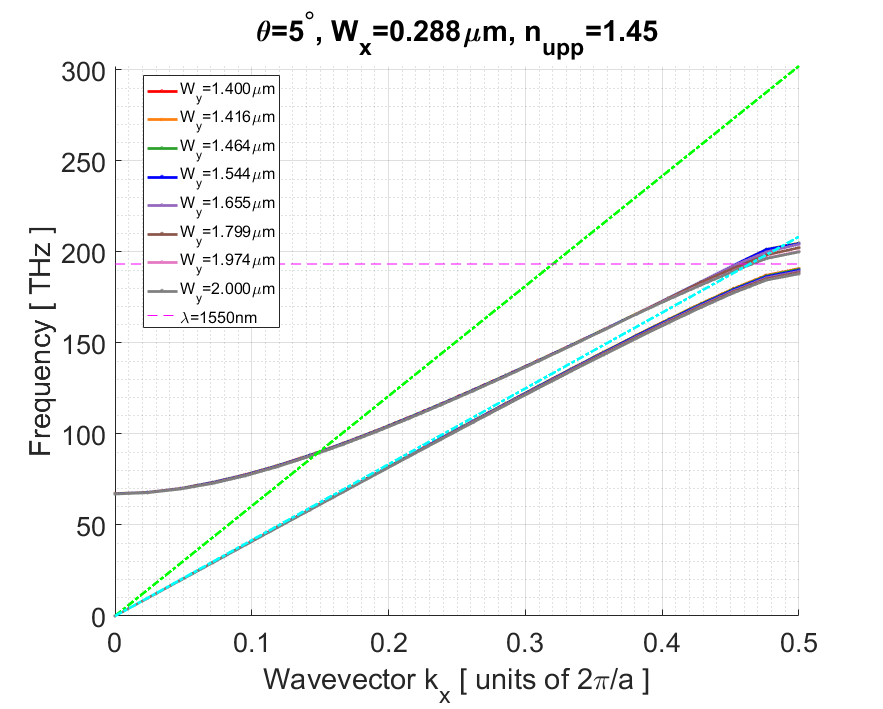


Figure S.23 – Calculated bandgaps corresponding to the PhCs with increasing stick height ($W_{y}$), from 1.4 µm to 2 µm. The green dashed line corresponds to the air light-lines.

Finally, the optical mode confinement and Q-factor behaviour of PhC cavities with asymmetric cladding and sidewall angles are strongly dependent on TE-TM mode coupling, which is typically a dominant loss mechanism, for which the optical performances of the PhC cavity exhibit asymmetric peaks with cladding RI [66,67].
